# Zn···Zn interactions at nickel and palladium centers†
†Electronic supplementary information (ESI) available. CCDC 1478225–1478229. For ESI and crystallographic data in CIF or other electronic format see DOI: 10.1039/c6sc02106a



**DOI:** 10.1039/c6sc02106a

**Published:** 2016-06-23

**Authors:** Kerstin Freitag, Mariusz Molon, Paul Jerabek, Katharina Dilchert, Christoph Rösler, Rüdiger W. Seidel, Christian Gemel, Gernot Frenking, Roland A. Fischer

**Affiliations:** a Inorganic and Metalorganic Chemistry , Technical University Munich , D-85748 , Garching , Germany . Email: roland.fischer@tum.de ; Email: roland.fischer@ruhr-uni-bochum.de; b Inorganic Chemistry II – Organometallics & Materials , Ruhr-University Bochum , D-44780 , Bochum , Germany; c Department of Chemistry , Philipps University Marburg , D-35032 Marburg , Germany . Email: frenking@chemie.uni-marburg.de

## Abstract

The analogy between ZnR fragments and the hydrogen radical represents a fruitful concept in organometallic synthesis. The organozinc(ii) and -zinc(i) sources ZnMe_2_ (Me = methyl) and [Zn_2_Cp*_2_] (Cp* = pentamethylcyclopentadienyl) provide one-electron fragments ·ZnR (R = Me, Cp*), which can be trapped by transition metal complexes [L_*a*_M], yielding [L_*b*_(ZnR)_*n*_]. The addition of the dizinc compound [Zn_2_Cp*_2_] to coordinatively unsaturated [L_*a*_M] by the homolytic cleavage of the Zn–Zn bond can be compared to the classic oxidative addition reaction of H_2_, forming dihydride complexes [L_*a*_M(H)_2_]. It has also been widely shown that dihydrogen coordinates under preservation of the H–H bond in the case of certain electronic properties of the transition metal fragment. The σ-aromatic triangular clusters [Zn_3_Cp*_3_]^+^ and [Zn_2_CuCp*_3_] may be regarded as the first indication of this so far unknown, side-on coordination mode of [Zn_2_Cp*_2_]. With this background in mind the question arises if a series of complexes featuring the Zn_2_M structural motif can be prepared exhibiting a (more or less) intact Zn–Zn interaction, *i.e.* di-zinc complexes which are analogous to non-classical dihydrogen complexes of the Kubas type. In order to probe this idea, a series of interrelated organozinc nickel and palladium complexes and clusters were synthesized and characterized as model compounds: [Ni(ZnCp*)(ZnMe)(PMe_3_)_3_] (**1**), [Ni(ZnCp*)_2_(ZnMe)_2_(PMe_3_)_2_] (**2**), [{Ni(CN^*t*^Bu)_2_(μ_2_-ZnCp*)(μ_2_-ZnMe)}_2_] (**3**), [Pd(ZnCp*)_4_(CN^*t*^Bu)_2_] (**4**) and [Pd_3_Zn_6_(PCy_3_)_2_(Cp*)_4_] (**5**). The dependence of Zn···Zn interactions as a function of the ligand environments and the metal centers was studied. Experimental X-ray crystallographic structural data and DFT calculations support the analogy between dihydrogen and dizinc transition metal complexes.

## Abstract 2

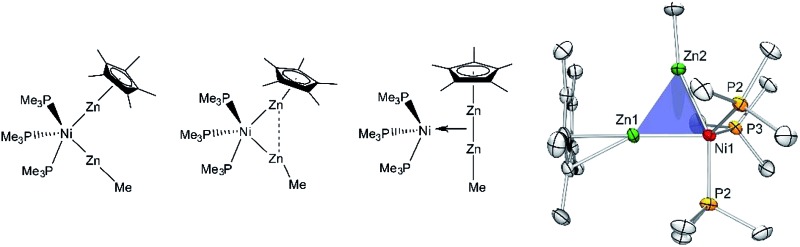
Zinc–zinc interactions on nickel and palladium centers are highly dependent on the co-ligands. These dependencies are also found for the formation of dihydrogen *vs.* dihydride complexes and underline the analogy [Zn_2_Cp*_2_] ↔ H_2_.

## 1 Introduction

Decamethyldizincocene, [Zn_2_Cp*_2_], a metalla-analogue to the dihydrogen molecule H_2_, was reported in 2004 by Carmona *et al.* as the first molecular compound with a covalent Zn^I^–Zn^I^ σ-bond, a bond that was rumored to be non-existent.^1^ [Zn_2_Cp*_2_] reveals a rich coordination chemistry. Reactions of [Zn_2_Cp*_2_] with transition metal complexes containing labile ligands L are typically based on an initial homolytic cleavage of the Zn–Zn bond, followed by ligand exchange, resulting in the coordination of ZnCp* fragments.^2–4^ As ZnCp* is isolobal to H, the most common reaction pattern of [Zn_2_Cp*_2_] towards transition metal centers L_*a*_M can be well compared to the “oxidative addition” reaction of H_2_ to electron-rich unsaturated transition metal fragments, forming dihydride complexes [L_*a*_M(H)_2_]. Very recently we observed a different coordination mode of [Zn_2_Cp*_2_] in the two triangular compounds [Zn_3_Cp*_3_]^+^ and [Zn_2_CuCp*_3_]. Both species are described as metalla-analogues of the σ-aromatic [H_3_]^+^ ion, again emphasizing the isolobal analogy between the fragment Cp*Zn˙ and the hydrogen atom H˙.^5^ In particular, we came across this conceptual analogy in 2008 with the discovery of [Mo(ZnMe)_9_(ZnCp*)_3_].^6^ This unique icosahedral coordinated complex can be regarded as a stable and accessible analogue of the matrix isolated polyhydride complex [WH_12_], a species which, differently from the [MoZn_12_] analogue, could better be written as [WH_6_(H_2_)_3_].^7^ The unique formation reaction of [Mo(ZnMe)_9_(ZnCp*)_3_] is based on the chemistry of homoleptic compounds [M(GaCp*)_*n*/2_] (*n* > 8; M = Mo, Ni, Pd, Pt, Cp* = pentamethylcyclopentadienyl) towards ZnMe_2_ or ZnEt_2_, which leads to pseudo homoleptic all organo-zinc coordinated products with the general formula [M(ZnR)_*n*_] (*n* ≥ 8; M = Mo, Ru, Rh, Ni, Pd, Pt; R = Cp*, Et, Me).^8^ This concept is transferable to heteroleptic starting materials [L_*m*_M(GaCp*)_*n*/2_] (L = Cp*, CO, X, PMe_3_…), where the ligands L have been able to control the reaction selectivity and product formation.^9–12^ A reaction pattern dependent on the co-ligands of a metal complex and thus the electronic properties of a system is also reported for the coordination of H_2_ to transition metal fragments. Not only are simple oxidative addition reactions, resulting in dihydride complexes known, the “side-on” coordination of H_2_ under the preservation of the H–H bond has also been widely studied and reported, especially by Kubas *et al.* ^13–15^ The triangular clusters [Zn_3_Cp*_3_]^+^ and [Zn_2_CuCp*_3_] can be regarded as the first examples with an H_2_ analogous “side-on” coordination mode of [Zn_2_Cp*_2_] at the two isoelectronic, unsaturated fragments [ZnCp*]^+^ and CuCp*.^5^ With this background in mind the question arises, if a series of complexes featuring the Zn_2_M structural motive can be prepared exhibiting a (more or less) intact Zn–Zn interaction, *i.e.* dizinc complexes which are analogous to non-classical dihydrogen complexes of the Kubas type.

## 2 Results and discussion

**Scheme 1:**
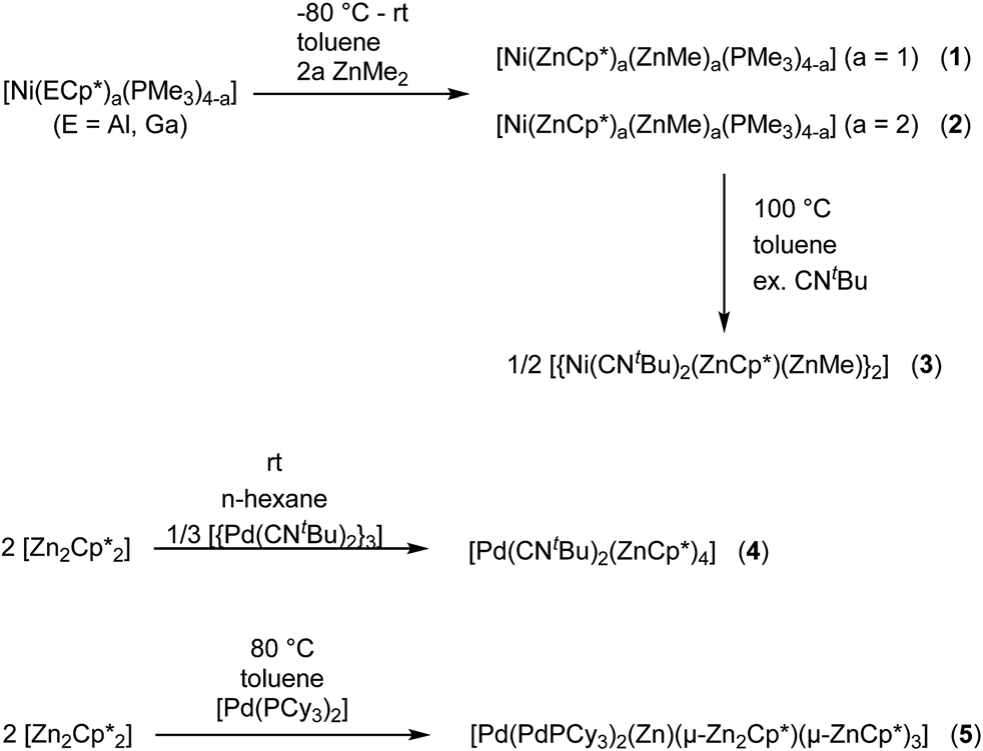
Synthesis scheme of the Ni/Zn compounds **1–3** and the Pd/Zn compounds **4** and **5**.

In this contribution, phosphine and isonitrile ligated heteronuclear Ni/Zn and Pd/Zn complexes with different L/Zn ligand ratios are described (Scheme 1). The compounds [Ni(ZnCp*)(ZnMe)(PMe_3_)_3_] (**1**) and [Ni(ZnCp*)_2_(ZnMe)_2_(PMe_3_)_2_] (**2**) are formed by a selective E/Zn exchange reaction when treating the heteroleptic starting materials [Ni(PMe_3_)_*a*_(ECp*)_*b*_] with ZnMe_2_ (E = Ga, Al; Cp* = pentamethylcyclopentadienyl; *a* + *b* = 4). The treatment of **1** with CN^*t*^Bu, GaCp* and AlCp* yielded the Ni/Zn cluster compound [{Ni(CN^*t*^Bu)_2_(μ_2_-ZnCp*)(μ_2_-ZnMe)}_2_] (**3**). Furthermore, reaction of [Zn_2_Cp*_2_] with [{Pd(CN^*t*^Bu)_2_}_3_] and [Pd(PCy_3_)_2_] (Cy = cyclohexyl) leads to the Pd/Zn compounds [Pd(ZnCp*)_4_(CN^*t*^Bu)_2_] (**4**) and [Pd_3_Zn_6_(PCy_3_)_2_(Cp*)_4_] (**5**). All new compounds **1–5** were characterized by NMR and IR spectroscopy, Liquid Injection Field Desorption Ionization (LIFDI) MS analysis, single crystal X-ray diffraction and elemental analysis. The electronic structures of **1** and **2** were investigated by quantum chemical calculations at the DFT level of theory.

### 2.1 Synthesis and spectroscopic characterization of **1** and **2**

The treatment of the heteroleptic Ni/(Al,Ga) starting compounds [Ni(ECp*)_*a*_(PMe_3_)_4–*a*_] (*a* = 1, 2; E = Ga, Al) with exact stoichiometric amounts ZnMe_2_ (1.2 M solution) in toluene as the solvent leads to [Ni(ZnCp*)(ZnMe)(PMe_3_)_3_] (**1**) and [Ni(ZnCp*)_2_(ZnMe)_2_(PMe_3_)_2_] (**2**), independent of the element E used in the starting complexes. Both compounds were isolated in good yields of 67% (**1**) and 84% (**2**). E^III^ containing by-products, Cp*EMe_2_ species, were observed. They are well soluble in organic solvents like benzene, toluene, or *n*-hexane and are stable for several weeks in the pure crystalline form when stored under argon at –30 °C. Yellow crystals of both compounds suitable for single crystal X-ray analysis were obtained from saturated hexane or toluene solutions, respectively. The elemental compositions were determined by combustion analysis (C, H) and atomic absorption spectroscopy (Zn), respectively. The empirical formulas were confirmed by mass spectrometric analyses using LIFDI, which show exclusively the presence of the molecular ion peaks at 568.09 *m*/*z* for 1 and 771.94 *m*/*z* for 2. The fine structure of the signals also match very well the calculated isotopic patterns. ^1^H NMR studies are also in agreement with the determined molecular structures in the solid state (see the Experimental section for details on ^31^P and ^13^C NMR studies). In contrast, analogous reactions using the sterically more bulky phosphine ligands PPh_3_ or PCy_3_ lead to a mixture of homoleptic products [Ni(ZnCp*)_4_(ZnMe)_4_] and [Ni(PR_3_)_4_] (R = Ph, Cy) in non-stoichiometric reactions. The observed facial coordination of three PR_3_ and two ZnR ligands in penta-coordinated **1** and two *cis*-PR_3_ and four ZnR in hexa-coordinated **2** is obviously favored only for the less bulky ligands PR_3_. It should be noted that the missing member of the employed series of starting compounds, [Ni(GaCp*)_3_(PR_3_)], is only isolable for R = Cy; not for R = Me.^16^ Thus, the monophosphine-substituted, hepta-coordinated complex [Ni(ZnCp*)_3_(ZnMe)_3_(PMe_3_)] remains unknown so far, pointing to very delicate, small kinetic effects related to steric overcrowding. For the larger central metal atom Pd, the all-zinc ligated hepta-coordinated [Pd(ZnCp*)_4_(ZnMe)_2_{Zn(tmeda)}]^17^ (tmeda = *N*,*N*,*N*′,*N*′-tetramethyl-ethane-1,2-diamine) is known, however. Here, six Zn atoms (acting as single electron ligands similar to ZnR in **1** and **2**) are arranged in an ideal trigonal dodecahedron way, where the {Zn(tmeda)} moiety is located almost exactly between two “missing” vertices of the dodecahedron (*S*_Q_(*P*) = 0.07, see ESI† for details of CShM). Conceptually, the {Zn(tmeda)} unit can be viewed as a two electron donor ligand with a steric bulk at least comparable to phosphine PR_3_ or ECp*. Therefore, the existing [Pd(ZnCp*)_4_(ZnMe)_2_{Zn(tmeda)}] and the so far still missing link [Ni(ZnCp*)_3_(ZnMe)_3_(PMe_3_)] belong to the same family of hepta-coordinated, heteroleptic complexes [M(ZnR)_6_L] (M = Ni, Pd, Pt; L = 2 electron donor ligand).

### 2.2 Molecular structures of **1** and **2** in the solid state

**Fig. 1:**
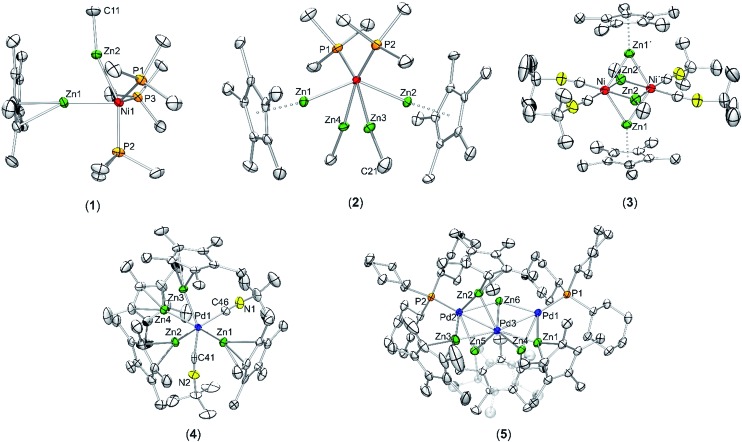
Molecular structure of compounds **1–5** in the solid state as determined by single crystal X-ray diffraction (thermal ellipsoids are shown at the 50% probability level, hydrogen atoms have been omitted for clarity). Selected bond length (Å), and angles (°): (**1**) Ni1–Zn1 2.351(1), Ni1–Zn2 2.309(1), Ni1–P1 2.159(1), Ni1–P2 2.146(1), Ni1–P3 2.159(1), Zn1–Zn2 2.525(1), Zn1–Ni1–Zn2 65.60(1), Ni–Zn2–Me 160.01(1), Ni–Zn1–Cp*_centroid_ 175.08, Cp*_centroid_–Zn1–Zn2 118.76, Me(C11)–Zn2–Zn1 141.91(1). (**2**) Ni1–Zn1 2.335(1), Ni1–Zn2 2.326(1), Ni1–Zn3 2.285(1), Ni1–Zn4 2.289(1), Ni1–P1 2.151(1), Ni1–P2 2.154(1), Zn1–Zn3 2.716(1), Zn1–Zn4 2.803(1), Zn2–Zn3 2.779(1), Zn2–Zn4 2.723(1), Zn3–Zn4 2.767(1), Zn1–Cp*_centroid_ 1.969, Zn2–Cp*_centroid_ 1.966; Ni–Zn1–Cp*_centroid_ 171.02, Ni–Zn1–Cp*_centroid_ 172.86, Ni1–Zn3–Me 171.11(2), Ni1–Zn4–Me 171.39(2), Zn1–Ni1–Zn2 137.60(3), Zn3–Ni1–Zn4 74.46(3). (**3**) Ni1–Ni1′ 2.572(1), Ni1–Zn1 2.418(1), Ni1–Zn1′ 2.438(1), Ni1–Zn2′ 2.392(1), Ni1–Zn2 2.400(1), Zn1–Zn2 2.953(1), Zn1–Zn2′ 2.817(1). (**4**) Pd–Zn1 2.484(1), Pd–Zn2 2.428(1), Pd–Zn3 2.439(1), Pd–Zn4 2.468(1), Zn1–Zn2 2.595(2), Zn3–Zn4 2.609(2), Pd–C46 2.019(1), Pd–C41 2.034(1), Zn1–Pd–Zn2 63.76(3), Zn3–Pd–Zn4 64.24(3), Pd–Zn1–Cp*_centroid_ 147.28, Pd–Zn2–Cp*_centroid_ 144.63, Pd–Zn3–Cp*_centroid_ 144.22, Pd–Zn4–Cp*_centroid_ 150.17. (**5**) Pd1–Pd3 2.723(1), Pd2–Pd3 2.669(1), Pd1–Zn1 2.551(1), Pd3–Zn1 2.563(1), Pd3–Zn2 2.555(1), Pd2–Zn2 2.628(1), Pd2–Zn3 2.531(1), Pd3–Zn3 2.555(1), Pd3–Zn4 2.457(1), Pd1–Zn4 2.471(1), Pd2–Zn5 2.498(1), Pd3–Zn5 2.478(1), Pd1–Zn6 2.512(1), Pd2–Zn6 2.504(1), Pd3–Zn6 2.494(1), Pd1–P1 2.309(1), Pd2–P2 2.307(1), Zn4–Zn5 2.729(1), Pd1–Pd2–Pd3 115.22(2).

Nickel adopts penta-and hexa-coordination in **1** and **2**, respectively, with different ratios of ZnR (R = Cp*, Me) and phosphine ligands according to Fig. 1. In both cases phosphine and organo-zinc ligands are arranged *fac* (**1**) and *cis* (**2**) and the structures are best described as composed of a tetrahedron (NiP_4_), in which one and two vertices are substituted by Zn_2_ units, respectively. For **2** this results in a polyhedron which consists of half of a tetrahedron (NiP_2_) and half of a dodecahedron (NiZn_4_) (see ESI† for CShM values). All Ni–Zn and Ni–P distances are comparable to the distances found in related compounds, *e.g.* [Ni(ZnCp*)_4_(ZnMe)_4_] (2.313(1)–2.371(1) Å for Ni–Zn) or [Ni(GaCp*)_2_(PMe_3_)_2_] (Ni–P: 2.139(1) Å).^8^ The P–Ni–P angles all lie between 106.12(2) and 121.10(3)°, which is rather close to the ideal tetrahedral angle of 109.5°.

### 2.3 Ligand exchange reactions at **1** and **2**

Substitution reactions of **1** or **2** with P(OMe)_3_ lead to a mixture of [Ni(P(OMe)_3_)_*x*_(PMe_3_)_(4–*x*)_] as major products, as determined by ^31^P and ^1^H NMR spectroscopy. Also dppe (1,2-bis(diphenylphospino)ethane) leads to the homoleptic complex [Ni(dppe)_2_] as the only identifiable compound. Likewise, the reactions with GaCp* or AlCp*, respectively, lead to undefinable product mixtures in all cases. However, treatment of **2** with *tert*-butylisonitrile at 100 °C in toluene yields the new Ni/Zn compound [{Ni(CN^*t*^Bu)_2_(μ_2_-ZnCp*)(μ_2_-ZnMe)}_2_] (**3**) (Scheme 1), besides liberation of Cp*ZnMe (1.98 and –0.65 ppm; see Fig. S1 of ESI†) and precipitation of metallic zinc (Table 1).

**Table 1:** Ligand exchange reactions with **1** and **2**

Ligand	Products, byproducts
P(OMe)_3_	[Ni(PR_3_)_4_] (R = OMe, Me), Cp*ZnMe, Zn
dppe^*a*^	[Ni(dppe)_2_], Cp*ZnMe, Zn
CN^*t*^Bu	[{Ni(CN^*t*^Bu)_2_(μ_2_-ZnCp*)(μ_2_-ZnMe)}_2_], Cp*ZnMe, Zn^#^
GaCp*/AlCp*	Decomposition

^*a*^dppe = 1,2-bis(diphenylphospino)ethane, ^#^ no formation of Cp*ZnMe and Zn with **1** as starting material.

From a conceptual point of view, the substitution of PMe_3_ by *tert*-butylisonitrile in **2** triggers elimination of Cp*ZnZnMe similar to a classic reductive elimination reaction, forming the (hypothetical) 16 valence electron (ve) fragment [Ni(CN^*t*^Bu)_2_(ZnMe)(ZnCp*)], which subsequently dimerizes to yield **3**. The heteroleptic organozinc(i) species Cp*ZnZnMe is thermally very unstable and thus spectroscopically not observable, and presumably disproportionates to yield the observed products, elemental Zn and Cp*ZnMe. Attempts to trap the monomeric 16ve species [Ni(CN^*t*^Bu)_2_(ZnMe)(ZnCp*)] by addition of excess PPh_3_ failed. As expected, **1** can be used as a starting material for the formation of **3** too, and with PMe_3_ as the only observable byproduct. The composition of **3** has been confirmed by elemental analysis and LIFDI-MS which shows the molecular ion peak at 1009.7 *m*/*z* exclusively (calcd 1010.2 *m*/*z*).

### 2.4 Molecular structure of **3** in the solid state

The core moiety of [{Ni(CN^*t*^Bu)_2_(μ_2_-ZnCp*)(μ_2_-ZnMe)}_2_] (**3**) can be described as a compressed Ni_2_Zn_4_ octahedron, where the Ni atoms are located *trans* along the short axis (Fig. 1). Both Ni sites are additionally coordinated by two *tert*-butylisonitrile ligands with a coplanar arrangement of the two Ni centers, Zn2, Zn2′ and all four CN moieties. Interestingly, despite a different ligand environment and a different cluster valence electron count (cve), the Ni_2_Zn_4_ core structure is very similar to that of the related complex [Ni_2_Zn_4_Cp_6_].^18^ The Ni–Ni distance in both clusters is almost equal with values of 2.572(1) Å in **3** and 2.571(2) Å in [Ni_2_Zn_4_Cp_6_]. The Ni–Zn (2.392(1)–2.438(1) Å) and Zn–Zn distances (2.953(1) and 2.817(1) Å) in **3** are also well comparable to those in [Ni_2_Zn_4_Cp_6_] (Ni–Zn: av. 2.40 Å, Zn–Zn: av. 2. Å) or [Ni(ZnCp*)_4_(ZnMe)_4_] (Ni–Zn: 2.313(1)–2.371(1) Å, Zn–Zn: 2.746(1)–2.912(1) Å). The terminal isonitrile ligands are slightly bent with Ni–C–N angles of 168.5(3) and 169.1(3)°, as well as C–N–C angles of 158.0(4) and 168.5(4)°. The deviation from linearity can be explained by the electron-rich situation at the nickel centers leading to strong π-back-bonding. Also weak interactions of the CN groups with the adjacent ZnMe ligands (C–C distances of 2.554(3) and 2.578(3) Å) cannot be excluded.

### 2.5 Synthesis, spectroscopic and structural characterization of **4**

Treatment of [{Pd(CN^*t*^Bu)_2_}_3_] with [Zn_2_Cp*_2_] in *n*-hexane at room temperature leads to the formation of [Pd(CN^*t*^Bu)_2_(ZnCp*)_4_] (**4**) as an orange microcrystalline solid. Recrystallization from toluene at –30 °C leads to yellow, cubic crystals suitable for single crystal X-ray diffraction. The empirical formula of **4** was derived from elemental analysis (C, H) and atomic absorption spectroscopy (Zn), respectively, and is consistent with the spectroscopic and structural data. The ^1^H NMR of compound **4** exhibits one signal for the *tert*-butylisonitrile methyl groups (*δ* = 1.18, s, 18H) as well as two signals for chemically inequivalent Cp* groups (*δ* = 2.10, s, 30H; *δ* = 2.27, s, 30H), which points to *cis*-coordination of the ^*t*^BuNC groups in the hexa-coordinate complex, matching with X-ray structural data (Fig. 1, below). The ^13^C NMR is also consistent with the suggested structure. The C–N and C≡N vibration bands in the FTIR spectrum can be observed at wavenumbers of 1188 and 2092 cm^–1^. Compound **4** crystallizes in the orthorhombic space group *Pbca* with *Z* = 4 with two independent molecules in the asymmetric unit. As both molecules are virtually the same in terms of their bond length and angles only one of them is discussed here (Fig. 1, below). The six ligands are coordinated in a strongly distorted octahedral arrangement to the palladium center. The Pd–Zn distances are all between 2.428(1)–2.484(1) Å and well comparable with other Pd-ZnCp* units known in literature.^2,8^ Similarly the Pd-CN^*t*^Bu bond lengths are all in a similar range to other palladium–isonitrile complexes.^9^

### 2.6 Synthesis, spectroscopic and structural characterization of **5**

Treatment of [Pd(PCy_3_)_2_] with two molar equivalents of [Zn_2_Cp*_2_] in 5 mL toluene leads to a red solution which after heating to 80 °C for one hour and standard workup gives [Pd(PdPCy_3_)_2_(Zn)(μ-Zn_2_Cp*)(μ-ZnCp*)_3_] (**5**) as dark red/black cubic single crystals. The elemental analysis (C, H) and atomic absorption spectroscopy (Zn) data, respectively, are consistent with the composition derived from single crystal X-ray diffraction studies (Fig. 1). Also, the ^1^H, ^13^C and ^31^P NMR spectra exhibit the expected signals, matching the molecular structure determined in the solid state (*vide infra*). The FTIR spectrum of **5** shows absorption bands for the C–H valence vibrations of the Cp* groups (*ν* = 2898 and 2827 cm^–1^) as well as a *p*-cylcohexyl vibration (*ν* = 1433 cm^–1^). Compound **5** crystallizes in the triclinic space group *P*1. The core of complex **5** is a [Pd_3_Zn_6_] unit, in which the three palladium atoms are adopting a bent structure with a Pd1–Pd3–Pd2 angle of 115.22(2)°. The Pd–Pd distances are 2.669(1) and 2.723(1) Å, which is in the range of other Pd–Pd distances in *e.g.* [Pd_2_(μ-GaCp*)_3_GaCp*)_2_] (2.609(1) Å)^19^ or [Pd_2_Zn_6_Ga_2_(Cp*)_5_Me_3_] (2.668 Å).^20^ Three of the six zinc atoms (Zn1, Zn2, Zn3) are ZnCp* units which are located in Pd–Pd bridging positions. Two Zn atoms (Zn4, Zn5) form a {Zn_2_Cp*} unit, which coordinates in a symmetric fashion to the Pd_3_ unit in such way that Zn4 is found bridging Pd1 and Pd3, and Zn5 bridging Pd2 and Pd3, with almost equal Pd–Zn distances (Pd3–Zn4 2.457(1), Pd1–Zn4 2.471(1), Pd2–Zn5 2.498(1), Pd3–Zn5 2.478(1)). The Cp* ring of the {Zn_2_Cp*} unit is disordered and thus binds asymmetrically to Zn4 and Zn5, the Zn–C bond distances suggesting η^1^–η^3^-binding modes for the two identical isomers. The Zn4–Zn5 distance (2.729(1) Å) in this unit is comparable to the Zn–Zn distance found in compound **2** which again may indicate weak Zn···Zn interactions. With the exception of Zn4–Zn5, all Zn–Zn distances are longer than 2.83 Å and thus outside the range of bonding Zn–Zn interactions. The last Zn atom (Zn6) is not coordinated by an organic ligand and is found in a position with an almost equal distance to all three palladium atoms (Pd1–Zn6 2.512(1), Pd2–Zn6 2.504(1), Pd3–Zn6 2.494(1)). All Pd–Zn distances (2.471(1)–2.563(1) Å) are elongated as compared to the terminally coordinated ZnR groups (R = Me, Cp*) in **4** (2.428(1)–2.484(1) Å) and [Pd(ZnMe)_4_(ZnCp*)_4_] (2.417(1)–2.459(1) Å)^8^ but well in the range of other ZnR ligands found in bridging positions.^10^ The central atom (Pd3) is located in the center of a pseudo-hexagon with one vacant vertex, consisting of alternating zinc and palladium atoms. Thus, Zn1 (0.186 Å), Zn3 (0.336 Å) and Zn6 (0.093 Å) as well as all three palladium atoms are almost perfectly coplanar with deviations from the plane of 0.186 Å (Zn1), 0.336 Å (Zn3) and 0.093 Å (Zn6). The bond angles, however, strongly deviate from those of a perfect hexagon.

## 3 Discussion of Zn···Zn interactions in compounds **1–5**

### 3.1 Structural comparisons

Our discussion whether two adjacent Zn atoms in compounds **1–5** show a significant Zn–Zn interaction or not is based in the first place on the evaluation of Zn–Zn distances as extracted from X-ray single crystal structure data – computational studies will complement the picture (*vide infra*). The distance of two Zn atoms in the (distorted) hexagonal closest packed structure of metallic zinc (Table 2), *i.e.* the metallic radius of zinc (1.339 Å),^21^ respectively, may serve as a point of reference. Distances above 2.66 Å may thus be regarded as non-or only weakly interacting. This is in good agreement with the distances found in the series of pseudo-homoleptic reference complexes [M(ZnCp*)_4_(ZnMe)_4_] (M = Ni, Pd, Pt) with values of 2.746(1) (M = Ni) and 2.824(1) Å, respectively.^8^ Theoretical examination of the bonding situation and analysis of the molecular orbitals in these compounds indeed showed only very weak Zn–Zn interactions which are merely sufficient to minimize ligand–ligand repulsion in these highly coordinated coordination compounds.

**Table 2:** Zn–Zn distances found in **1–5** as well as in some reference compounds

Compound	Shortest Zn–Zn Distance/Å
[Zn_2_Cp*_2_]^1^	2.305(3)
[Ni(ZnCp*)(ZnMe)(PMe_3_)_3_] (**1**)	2.525(1)
[Ni(ZnCp*)_2_(ZnMe)_2_(PMe_3_)_2_] (**2**)	2.718(1)
[{Ni(CN^*t*^Bu)_2_(ZnCp*)(ZnMe)}_2_] (**3**)	2.817(1)
[Pd(CN^*t*^Bu)_2_(ZnCp*)_4_] (**4**)	2.595(2)
[Pd_3_Zn_6_(PCy_3_)_2_(Cp*)_4_] (**5**)	2.723(1)
[Zn_3_Cp*_3_]^+^ (ref. 5)	2.430(1)
[Zn_2_CuCp*_3_]^5^	2.357(1)
[Ni(ZnCp*)_4_(ZnMe)_4_]^8^	2.746(1)
[Pd(ZnCp*)_4_(ZnMe)_4_]^8^	2.824(1)
Zinc(hcp)^22^	2.6636(1)

**Scheme 2:**
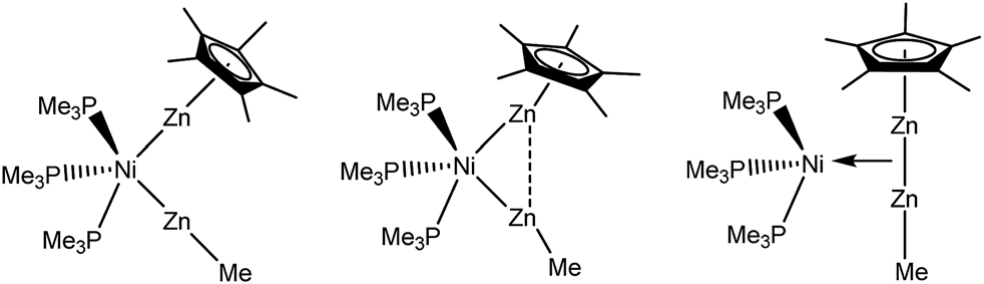
Different bonding modes of the Zn_2_R_2_ groups. Left: no Zn···Zn interaction, middle: weak Zn···Zn interaction, right: side on bonded Zn_2_R_2_ moiety, with significant Zn···Zn interaction.

The distance found in [Zn_2_Cp*_2_] (2.305(3) Å)^1^ may serve as a second point of reference, representing the minimum Zn–Zn distance which could be found in organozinc complexes or clusters. The Zn(i) dimer exhibits a Zn–Zn single (σ-)bond together with considerable participation of electrostatic attractions.^23^ Indeed, the recently published triangular clusters [Zn_3_Cp*_3_]^+^ (2.430(1) Å) and [Zn_2_CuCp*_3_] (2.357(1) Å) both show Zn–Zn distances in between the two extremes of metallic zinc and the Zn–Zn σ-bond in [Zn_2_Cp*_2_]. In both clusters, the three metal atoms are held together by two delocalized electrons. Theoretical investigation of the bonding situations in these molecules hinted at σ-aromaticity. However, in an alternative point of view the triangular structures may be also regarded as coordination compounds of [Zn_2_Cp*_2_] to the fragments [ZnCp*]^+^ or [CuCp*], respectively. In a way, this situation is similar to the coordination of H_2_ to transition metal fragments: unsaturated electron-rich metal fragments usually interact with the H_2_ molecule giving a dihydride species by oxidative cleavage of the hydrogen–hydrogen bond (“classical dihydride complexes”), while electron-poor unsaturated metal fragments lead to the formation of H_2_-adduct-complexes, without cleavage of the hydrogen–hydrogen bond (“non-classical” or “Kubas-type dihydrogen complexes”).^14^ However, while the oxidative addition of H_2_ includes oxidation of the transition metal center, the addition of [Zn_2_Cp*_2_] to metal fragments proceeds without formal change of oxidation state. The distinction between pure Zn_2_R_2_ coordination without Zn–Zn-bond cleavage and products with distinct ZnR ligands exhibiting no Zn–Zn interaction is not solely based the Zn–Zn distances as discussed above, but also other structural parameters can be used: The Zn–Zn–R as well as M–Zn–R bond angles for instance are important indicators for the presence of Zn–Zn interactions. While a strictly side-on coordinated Zn_2_R_2_ should ideally exhibit a linear R–Zn–Zn–R geometry (not regarding steric effects in the first place), the Zn–Zn–R bond angles gradually decrease towards 120° for perfectly symmetric MZn_2_R_3_ complexes with a weaker Zn–Zn interaction. At the same time, the M–Zn–R bond angles increase towards 180° (Scheme 2).

**Fig. 2:**
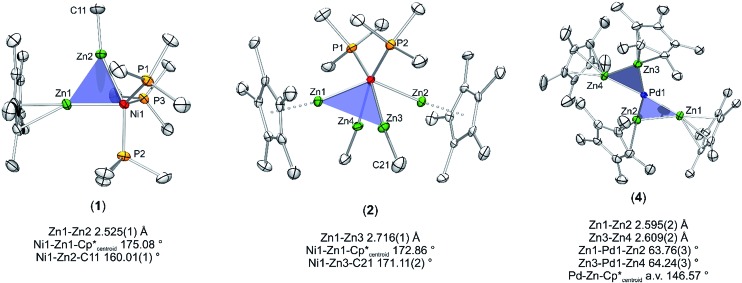
Triangular motifs in compounds **1**, **2** and **4**. The *tert*-butylisonitrile ligands in compound **4** are omitted for clarity.

With this background in mind, it is worth to have a closer look at the Zn–Zn distances measured for compounds **1–4**. Most significant is the short distance between the Zn atoms in **1** (2.525(1) Å), which is 13% shorter as compared to the higher coordinated [Ni(ZnCp*)_4_(ZnMe)_4_] (2.746(1) to 2.912(1) Å). For the latter complex very weak Zn···Zn interactions were proposed, however, with no direct Zn–Zn bond paths. The covalent Zn–Zn single bond length in [Zn_2_Cp*_2_] of 2.305(3) Å, which may serve as a reference for a strong Zn–Zn σ-bond, is only 9% shorter than the one observed in **1**. Obviously, the Zn–Zn distance of compound **1** lies almost exactly in between a weak tangential Zn–Zn interaction in the cluster-like compound [Ni(ZnCp*)_4_(ZnMe)_4_] and the classical, unsupported σ-bond in [Zn_2_Cp*_2_] (see Table 2). Similar to **1**, the shortest Zn–Zn distances in **4** (2.595(2) Å for Zn1–Zn2 and 2.609(2) Å for Zn3–Zn4) are only 12–13% longer than the covalent Zn–Zn interaction in [Zn_2_Cp*_2_].^1^ All other Zn–Zn distances in **4** are much longer (3.093–3.753 Å) and outside the range of weak Zn–Zn interactions. The Zn···Zn contacts in **1** and **4** are shorter than in metallic zinc (2.664 Å for the closest Zn–Zn distances).^22^ In contrast to **1** and **4** the shortest Zn–Zn distances in complexes **2** (2.718(1) Å) and **3** (2.817(1) Å) are outside the expected range for bonding Zn–Zn interactions and similar to those found in [Ni(ZnCp*)_4_(ZnMe)_4_]. Thus, based on the above comparisons, the Zn···Zn interactions follow the trend **1** ≈ **4** < **2** < **3** ≈ [Ni(ZnCp*)_4_(ZnMe)_4_]. The assumption of two significant Zn···Zn interactions in **1** and **4** is further supported by the respective bond angles: The M–Zn–Cp*_centroid_ angle for **1** (175.08°) deviates only slightly from linearity, while those of **4** (144.22–150.17°) are indeed closer to 150°, as expected for a perfectly symmetric M_3_ triangle.^5^ While in the case of **4** steric repulsion of the Cp* rings, which might influence the M–Zn–Cp*centroid angle, cannot be excluded, no steric Cp*/Cp* repulsion is present in 1 (see Fig. S11 and S12† for the depiction of space filling models of 1 and 4). As consequence of the bent structure and the involved steric repulsion, the Cp* rings of **4** are not η^5^ coordinated to the Zn centers but are closer to η^2^ (Zn2), η^3^ (Zn1, Zn3) and η^4^ (Zn4) bonding modes. Furthermore, the Zn1–Pd–Zn2 (63.76(3)°) and Zn3–Pd–Zn4 (64.24(3)°) angles of **4** are also clearly closer to a triangular geometry (60°) than an octahedral geometry (90°) (Fig. 2).

### 3.2 Quantum chemical investigations for compounds **1** and **2**

**Fig. 3:**
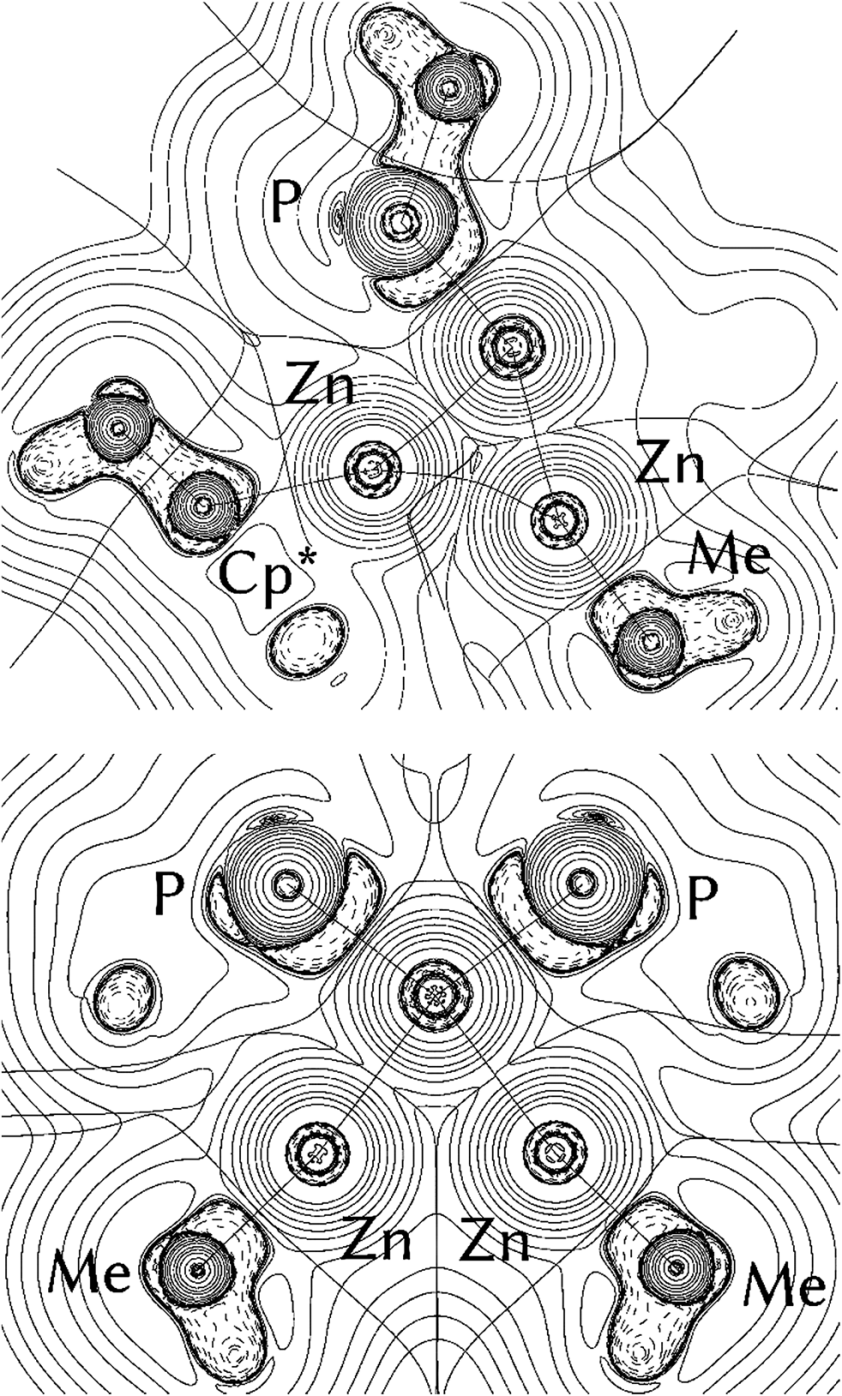
Contour line diagrams ∇^2^ *ρ*(*r*) of **1** (top) and **2** (bottom). Solid lines indicate areas of charge concentration (∇^2^ *ρ*(*r*) < 0) while dashed lines show areas of charge depletion (∇^2^ *ρ*(*r*) > 0). The thick solid lines connecting the atomic nuclei are the bond paths. The thick solid lines separating the atomic basins indicate the zero-flux surfaces crossing the molecular plane.

In order to answer the question if there are significant differences between the metal–ligand interactions of the ZnR ligands in **1** and **2**, we first optimized the geometries of the complexes at the BP86/TZVPP level. After that, QTAIM calculations, natural bond orbitals (NBO) and energy decomposition analyses with the “Natural Orbitals for Chemical Valence” extension (EDA-NOCV) were performed to get insight into the bonding situation of the adducts. The optimized structures of **1** and **2** (Fig. S13 and S14†) are in good agreement with the experimental data given by single crystal X-ray diffraction measurements. The calculated bond lengths are generally about 0.040 Å too large compared to experiment, with the exception being the Ni–ZnCp* bonds where the deviation is about 0.080 Å. The differences can be explained with packing effects in the crystal which are absent in the gas phase. The topological QTAIM analysis of **1** shows a bond path between the zinc atoms (Fig. 3, top) while there are no Zn–Zn bond paths in **2** (Fig. 3, bottom). This suggests a Zn–Zn bond in **1** which is absent in **2**. Although it is possible that there are interactions between atoms without bond paths according to the QTAIM,^30^ the very short Zn–Zn distance in **1** strongly supports the interpretation of an [Zn_2_R_2_] dimer interacting with [Ni(PMe)_3_]. The NBO analysis (Table S2†) predicts a Wiberg bond index (WBI) of 0.26 between the Zn atoms in **1**. This suggests a weaker Zn–Zn bond compared to [(ZnMe)(ZnCp*)] (WBI: 0.65), but the WBI value is still higher than for the Zn–Zn bond in **2** (0.07–0.15). Furthermore, the Ni–Zn bond in **1** appears weaker than in **2**, the WBI values being ≈0.25 and ≈0.55, respectively. This can be understood by considering the charge transferred from the ZnR ligands to Ni. While the nickel atom in **1** is only slightly charged (–0.23 e), it becomes distinctly more negative in **2** (–2.37 e). The data indicate that the Ni atom in **2** receives more electron density from the ZnR groups than in **1**. The EDA-NOCV results for **1** are given in Table S3.† The fragmentation into a [Zn_2_R_2_] dimer and [Ni(PMe)_3_] gives an intrinsic interaction energy of Δ*E*_int_ = –49.8 kcal mol^–1^. By visual inspection of the deformation densities, the two different contributions can be identified as part of the attractive orbital term Δ*E*_orb_: The donation of electron density [Zn_2_R_2_] → [Ni(PMe)_3_] gives an attractive interaction of –17.0 kcal mol^–1^, and the back donation [Zn_2_R_2_] ← [Ni(PMe)_3_] contributes –27.7 kcal mol^–1^ to the orbital interactions; thus, it is significantly stronger. The interacting fragments for the EDA-NOCV of **2** were chosen to be ZnR and [Ni(PMe)_3_(ZnR)_3_], which are in agreement with the QTAIM results of separated ZnR ligands in **2**. We carried out two calculations for **2** with the ligands ZnMe and ZnCp* as fragments, respectively. The results are very similar (Tables S4 and S5†). The total bond energy (Δ*E*_int_ = ≈–68.0 kcal mol^–1^) in both species is always higher compared to **1**. The main contribution of the orbital interactions is now the σ-type (ZnR) → [Ni(PMe)_3_(ZnR)_3_] donation which amounts to *ca.* 75% of Δ*E*_orb_. Note that the equivalent (ZnR) → [Ni(PMe)_3_(ZnR)_3_] donation in **2** is negligible. The bonding situation can be summarized as follows: There is a distinct Zn–Zn bond in compound **1**. The orbital interactions come mainly from the [Ni(PMe)_3_] → [Zn_2_R_2_] back donation, whereas the [Zn_2_R_2_] → [Ni(PMe)_3_] donation is roughly half as strong as the back donation. There is weaker Ni–Zn and stronger Zn–Zn bonding in **1** compared to 2 in which the ZnR groups can be seen as four separated (“classical”) electron donating ligands.

## 4 Conclusion

Our results show that Zn···Zn interactions in organozinc transition metal complexes are highly dependent on the co-ligands and metal centers. Whereas two phosphine ligands coordinated to a nickel centre mostly do not affect the Zn···Zn interaction, three phosphine ligands as in the case of [Ni(PMe_3_)_3_(ZnCp*)(ZnMe)] (**1**) lead to a significant Zn···Zn contraction to a distance of 2.523(1) Å, which is only about 9% longer than the covalent, unsupported σ-bond in [Zn_2_Cp*_2_]. Using a more electron-rich metal like palladium requires a relatively electron poor ligand sphere to preserve the Zn–Zn interaction of the parent compound [Zn_2_Cp*_2_]. In contrast, the use of the electron rich PCy_3_ as a ligand leads to cleavage of the Zn–Zn and coordination of ·ZnCp* fragments. Even the reduction to a formal Zn(0) centre and trapping of this during cluster formation can take place as shown by compound **5**. Very weak Zn···Zn interactions, in any, are present in such a case. These general dependencies from ancillary ligands L and metal centers M are also known for the formation of dihydrogen *vs.* dihydride complexes and are well comparable with our case here. Thus, we conclude that compound **1**, rewritten as [(Me_3_P)_3_Ni{η^2^-(Cp*ZnZnMe)}], may serve as an organozinc congener of the Kubas-type dihydrogen complex.^15^

## 5 Experimental

### 5.1 General remarks

All manipulations were carried out in an atmosphere of purified argon using standard Schlenk and glove box techniques. Hexane, toluene and THF were dried using a MBraun Solvent Purification System. The final H_2_O content in all solvents was checked by Karl Fischer titration and did not exceed 5 ppm. [GaCp*],^24^ [AlCp*],^25,26^, [Ni(PMe_3_)_2_(GaCp*)_2_],^16^, [Ni(PMe_3_)_3_(GaCp*)],^16^ [Zn_2_Cp*_2_],^27^, [{Pd(CN^*t*^Bu)_2_}_3_]^28^ and [Pd(PCy_3_)_2_]^29^ were prepared according to recent literature methods. Elemental analyses were performed by the micro analytical laboratory at the Ruhr-University Bochum, and Laboratory for Microanalytics at the University of Essen (EA 1110 CHNS-O Carlo Erba Instruments) and the Kolbe Laboratory for Microanalytics in Mülheim an der Ruhr. IR spectra were recorded on an ALPHA-T FT-IR spectrometer equipped with a single-reflection ATR sampling module operated in a glove box. NMR spectra were recorded on a Bruker Avance DPX-250 or a DPX-400 spectrometer (^1^H, 250.1 MHz; ^13^C, 62.9 MHz) in either C_6_D_6_ or C_7_H_8_ at various temperatures of 228 to 378 K. Chemical shifts are given relative to TMS and were referenced to the solvent resonances as internal standards. The chemical shifts are described in parts per million (ppm), downfield shifted from TMS and are consecutively reported as position (^1^H and ^13^C), relative integral, multiplicity (s = singlet, d = doublet, t = triplet, m = multiplet), coupling constant (*J* in Hz) and assignment.

### 5.2 [Ni(PMe_3_)_3_(ZnCp*)(ZnMe)] (**1**)

[Ni(PMe_3_)_3_(GaCp*)] (103 mg, 0.21 mmol) was suspended in toluene (4 mL) and cooled to –80 °C. Then ZnMe_2_ (0.35 mL of a 1.2 M solution in toluene) was added to the yellow suspension and stirred for 10 min. After 5 min the suspension turned into an orange solution during heating to RT and in the meantime the solvent was reduced *in vacuo*. The residue was dissolved in a small amount of toluene and single crystals were obtained at –30 °C within a few days. Yield: 67% (80 mg, 0.14 mmol). [Ni(PMe_3_)_3_(AlCp*)] can also be used as the starting material giving **1** in a poorer yield of 62%. ^1^H NMR (250.1 MHz, C_6_D_6_, 298 K): *δ* = 2.28 (s, 15H, ZnCp*), 1.07 (d, *J* = 3.7 Hz, 27H, PMe_3_), –0.04 (s, 3H, ZnMe). ^13^C NMR (62.9 MHz, C_6_D_6_, 298 K): *δ* = 109.5 (s, C_5_Me_5_), 27.0–26.1 (m, PMe_3_), 11.3 (s, ZnMe), 10.4 (s, C_5_Me_5_). ^31^P NMR (101.2 MHz, C_6_D_6_, 298 K): *δ* = –19.58 (s). Elemental anal. calcd for C_20_H_45_NiP_3_Zn_2_: C, 42.3; H, 8.0; Zn, 23.0; found: C, 42.1; H, 7.7; Zn, 22.5%.

### 5.3 [Ni(PMe_3_)_2_(ZnCp*)_2_(ZnMe)_2_] (**2**)

[Ni(PMe_3_)_2_(GaCp*)_2_] (450 mg, 0.73 mmol) was dissolved in toluene (5 mL) and cooled to –80 °C. Subsequently 2.42 mL (2.92 mmol) of a 1.2 M solution of ZnMe_2_ in toluene were added. The solution was heated to RT and stirred for 1 h. All volatile materials were evaporated *in vacuo*. The precipitate was washed with a small amount of cold hexane and the residue was finally dried *in vacuo* to yield a yellow powder. After storing a concentrated solution in hexane, twinned crystals could be obtained after a few days when stored at –30 °C. Other solvents and crystallization methods did not lead to single crystal formation, but twins were obtained in all cases. Yield: 84% (474 mg, 0.61 mmol). ^1^H NMR (250.1 MHz, C_6_D_6_, 298 K): *δ* = 2.20 (s, 30H, ZnCp*), 1.01 (d, 18H, PMe_3_), –0.02 (s, 6H, ZnMe). ^13^C NMR (62.9 MHz, C_6_D_6_, 298 K): *δ* = 109.6, 28.5, 28.2, 28.2, 28.0, 11.2 ppm. ^31^P NMR (101.2 MHz, C_6_D_6_, 298 K): *δ* = 9.03 (s). Elemental anal. calcd for C_28_H_54_NiP_2_Zn_4_: C, 43.5; H, 7.0; Zn, 33.8; found: C, 43.4; H, 7.4; Zn, 33.2%.

### 5.4 [{Ni(CN^*t*^Bu)_2_(μ_2_-ZnCp*)(μ_2_-ZnMe)}_2_] (**3**)

Method 1: **2** (200 mg, 0.26 mmol) was dissolved in toluene (5 mL) and an excess of CN*t*-Bu (10 eq. 0.28 mL, 2.60 mmol) was added. The reaction solution was heated to 100 °C overnight whereupon metal precipitation occurred. All volatile materials were evaporated *in vacuo* and the residue was extracted from toluene (2 × 3 mL) giving a clear orange solution, which gave after evaporation of all volatile materials an orange powder in a yield of 90% (116 mg, 0.12 mmol). Single crystals can be obtained from a saturated solution in toluene overnight when stored at –30 °C. Method 2: **1** (100 mg, 0.18 mmol) can also be used as the starting material, giving under the same reaction conditions no metal precipitation 82 mg (0.08 mmol) of an orange power in a yield of 89%. ^1^H NMR (250.1 MHz, C_6_D_6_, 298 K): *δ* = 2.30 (s, 30H, ZnCp*), 1.26 (s, 36H, CN*t*-Bu), 0.10 (s, 6H, ZnMe). ^13^C NMR (62.9 MHz, C_6_D_6_, 298 K): *δ* = 108.7, 55.7, 30.6, 11.0 ppm. Elemental anal. calcd for C_42_H_72_N_4_Ni_2_Zn_4_: C, 50.2; H, 7.2; N, 5.5; found: C, 50.1; H, 7.2; N, 5.5%.

### 5.5 [Pd(CN^*t*^Bu)(ZnCp*)_4_] (**4**)

130 mg [{Pd(CN^*t*^Bu)_2_}_3_] (0.040 mmol) and 384 mg [Zn_2_Cp*_2_] (0.956 mmol) were dissolved in 5 mL *n*-hexane after which the solution turned red. After 10 min of stirring, an orange, microcrystalline solid started to precipitate. The reaction mixture was stirred for one hour and, the solid was isolated *via* canula, washed with *n*-hexane (3 portions of 3 mL). After drying in *in vacuo*, the product was recrystallized in 6 mL toluene at –30 °C, giving yellow cubic crystals in yields around 31%. ^1^H NMR (250.1 MHz, C_6_D_6_, 298 K): *δ* = 2.27 (s, 30H, ZnCp*), 2.10 (s, 30H, ZnCp*), 1.18 (s, 18H, CN^t^Bu), ^13^C NMR (250.1 MHz, C_6_D_6_, 298 K): *δ* = 109.71 (s, *C*_5_Me_5_), 109.30, (s, *C*_5_Me_5_), 55.94 (s, CN*C*Me_3_), 30.54 (s, CNC*Me*_3_), 11.78 (s, C_5_ *Me*_5_) ppm. IR (cm^–1^): 2950, 2878, 2832, 2092, 1769, 1461, 1427, 1409, 1366, 1220, 1188, 1023, 851, 790, 724, 689, 603, 583, 498, 460. Elemental anal. calcd for C_50_H_78_N_2_PdZn_4_: C: 55.85, H 7.31, N 2.61, Pd 9.89, Zn 24.33; found: C 55.35, H 6.89, N 2.61, Pd 10.98, Zn 23.85.

### 5.6 [Pd_3_Zn_6_(PCy_3_)_2_(Cp*_4_)] (**5**)

100 mg [Pd(PCy_3_)_2_] (0.150 mmol) and 126 mg [Zn_2_Cp*_2_] (0.315 mmol) were dissolved in 5 mL toluene. The resulting red solution was heated to 80 °C for the period of one hour. After cooling to room temperature the dark red solution was reduced to 2 mL and stored at –30 °C. Dark red/black crystals, suitable for single crystal X-ray diffraction were obtained overnight. ^1^H NMR (250.1 MHz, C_6_D_6_, 298 K): 2.30 (d, 60H, C_5_Me_5_), 1.72 (m, 66H) ppm, ^31^P NMR (250.1 MHz, C_6_D_6_, 298 K): 52.26 ppm ^13^C NMR (250.1 MHz, C_6_D_6_, 298 K): 115.25 (s, *C*_5_Me_5_), 111.01 (s, *C*_5_Me_5_), 34.44 (PCy_3_), 32.14 (PCy_3_), 28.22 (PCy_3_), 27.20 (PCy_3_), 13.13 (C_5_ *Me*_5_), 12.45 (C_5_ *Me*_5_) ppm. IR (cm^–1^): 2898, 2827, 1643, 1597, 1433, 1343, 1265, 1111, 993, 925, 879, 831, 738, 722, 706, 688, 667, 614, 444. Elemental anal. calcd for C_86_H_134_P_2_Pd_3_Zn_6_ (5·C_7_H_8_): C 52.31, H 7.08, Pd 16.75, Zn 20.59; found: C 53.17, H 7.18, Pd 16.72, Zn 20.40.
