# Supplementary material for: Zn···Zn interactions at nickel and palladium centers
Source: Chem Sci. 2016 Jun 23;7(10):6413–21. doi: 10.1039/c6sc02106a (PMC5355958; doi:10.1039/c6sc02106a)
Supplement: Supplementary file 1 [file SC-007-C6SC01580H-s001.pdf]

Supporting Information

Zn···Zn interactions at Nickel and Palladium Centers

*Kerstin Freitag [a], Mariusz Molon [b], Paul Jerabek [c], Katharina Dilchert [a], Christoph Rösler [b], Rüdiger W. Seidel [b], Christian Gemel [a], Gernot Frenking [*c] and Roland A. Fischer* [a]*

[a] Inorganic and Metalorganic Chemistry, Technical University Munich, D-85748, Garching (Germany)

e-mail: roland.fischer@tum.de

[b] Inorganic Chemistry II - Organometallics & Materials, Ruhr-University Bochum, D-44780 Bochum (Germany), fax (+49)234 321 4174

[c] Department of Chemistry, Philipps University Marburg, D-35032 Marburg (Germany)

E-mail: frenking@chemie.uni-marburg.de

X-ray Crystallography: The X-ray intensity data for **1**, **2** and **4** · C_7H_8 were measured on an Oxford Diffraction Xcalibur2 diffractometer with a Sapphire2 CCD and graphite-monochromated Mo-K_α radiation. Those for **3** and **5** · 3 C_7H_8 were collected on an Agilent Technologies SuperNova diffractometer with an Atlas CCD and Cu-K_α radiation from multilayer X-ray optics. The crystals were coated with a perfluoropolyether, picked up with a glass fiber or a cryo loop and immediately mounted in the nitrogen cold gas stream of the diffractometer. The data were processed with the CrysAlisPro software [29]. Absorption corrections based on multiple-scanned reflection were carried out with ABSPACK in CrysAlisPro. The crystal structures were solved by direct methods with SHELXS-97 and refined with SHELXL-2014 [30].

The crystal of **2** was a non-merohedral twin with the twin operation corresponding to a twofold rotation about the [001] direction. The twin law is:

$$\begin{pmatrix} -1 & 0 & -0.0564 \\ 0 & -1 & 0 \\ 0 & 0 & 1 \end{pmatrix}$$

The twinning was taken into account by using the HKLF 5 option of SHELXL. Refinement of the fractional contributions of the two twin components yielded a ratio of 0.5891(6):0.4109(6). The crystal of **4** · C_7H_8 was twinned by pseudo-merohedry. The twin operation is a twofold rotation about the [$1\bar{1}0$] direction. The twin law is:

$$\begin{pmatrix} 0 & 1 & 0 \\ -1 & 0 & 0 \\ 0 & 0 & -1 \end{pmatrix}$$

The ratio of the twin components refined to 0.7792(7):0.2208(7). A Cp* ligand in **5** · 3 C_7H_8 and several toluene molecules in **4** · C_7H_8 and **5** · 3 C_7H_8 are disordered. Split models were refined with appropriate geometric restraints and rigid groups and restraints on atomic displacement parameters (ADPs). Anisotropic ADPs were introduced for all non-hydrogen atoms except for some disordered parts. Hydrogen atoms were placed at idealized posistions and refined with the appropriate riding model. Crystal data and refinement details for **1–3**, **4** · C_7H_8 and **5** · 3 C_7H_8 are listed in Table S1.

CCDC 1478226 (**1**), 1478227 (**2**), 1478225 (**3**), 1478229 (**4** · C_7H_8), 1478228 (**5** · 3 C_7H_8) contain the supplementary crystallographic data for this paper. These data can be obtained free of charge from the Cambridge Crystallographic Data Centre via www.ccdc.ac.uk/data_request/cif.

Table S1. Crystal data and refinement details for **1–3**, **4** · C_7H_8 and **5** · 3 C_7H_8.

	**1**	**2**	**3**	**4** · C_7H_8	**5** · 3 C_7H_8
empirical formula	$C_{20}H_{45}NiP_3Zn_2$	$C_{28}H_{54}NiP_2Zn_4$	$C_{42}H_{72}N_4Ni_2Zn_4$	$C_{57}H_{86}N_2PdZn_4$	$C_{97}H_{150}P_2Pd_3Zn_6$
M_r	567.92	772.84	1011.93	1167.15	2089.52
T (K)	107(2)	104(2)	102(2)	100(2)	100(2)
λ (Å)	0.71073	0.71073	1.54184	0.71073	1.54184
crystal size (mm^3)	0.56 × 0.36 × 0.27	0.25 × 0.19 × 0.13	0.08 × 0.05 × 0.03	0.26 × 0.24 × 0.23	0.11 × 0.09 × 0.08
crystal system, space group	triclinic, P-1	monoclinic, $P2_1/n$	monoclinic, $P2_1/n$	orthorhombic, $Pbca$	triclinic, P-1
a (Å)	9.2494(4)	11.8240(9)	11.9881(6)	25.6662(6)	12.2816(3)
b (Å)	10.0833(4)	17.8591(10)	17.8532(7)	25.5792(6)	13.0205(4)
c (Å)	15.9728(6)	16.3145(9)	12.4745(7)	35.0042(8)	29.8246(8)
α (°)	100.172(3)	90	90	90	100.247(2)
β (°)	92.322(3)	92.362(5)	109.769(6)	90	97.965(2)
γ (°)	109.339(4)	90	90	90	90.646(2)
V (Å^3)	1375.66(10)	3442.1(4)	2512.5(2)	22981.0(9)	4644.8(2)
Z	2	4	2	16	2
$\rho_{calc.}$ (mg m^{-3})	1.371	1.491	1.338	1.349	1.494
μ (mm^{-1})	2.585	3.392	3.120	1.988	6.886
F(000)	596	1600	1056	9728	2160
θ range for data collection (°)	2.992 - 28.929	2.859 - 29.167	4.423 - 75.079	2.864 - 28.882	3.452 - 74.188
index ranges	$-12 \le h \le 11$	$-15 \le h \le 15$	$-12 \le h \le 14$	$-34 \le h \le 33$	$-10 \le h \le 15$
	$-11 \le k \le 12$	$-23 \le k \le 23$	$-15 \le k \le 21$	$-32 \le k \le 33$	$-15 \le k \le 16$
	$-21 \le l \le 21$	$-21 \le l \le 20$	$-15 \le l \le 13$	$-43 \le l \le 46$	$-35 \le l \le 37$
reflections collected / unique	13105 / 6303	14063 / 14063	9131 / 4945	208231 / 28690	43311 / 18285
observed reflections [$I > 2\sigma(I)$]	5583	8985	3802	18902	15057
R_{int}	0.0290	-	0.0285	0.1063	0.0436
Parameters / restraints	250 / 0	317 / 0	247 / 0	1193 / 185	1058 / 364
goodness-of-fit on F^2	1.051	0.940	1.056	1.084	1.013
$R1$ [$I > 2\sigma(I)$]	0.0348	0.0370	0.0371	0.0585	0.0349
$wR2$ (all data)[a]	0.0961	0.0977	0.1054	0.1725	0.0910
Residuals (e Å^{-3})	1.224 / –0.793	1.142 / –0.563	0.884 / –0.492	2.340 / –1.122	1.167 –1.201

$R_1 = \sum \| F_{obs}| - |F_{cal}\| / \sum |F_{obs}|^{1/2}; wR_2 = \left[\sum w\left(F_{obs}^2 - F_{cal}^2\right) / \sum w\left(F_{obs}^2\right)^2\right]^{1/2}; GOF = \left\{\sum \left[w\left(F_{obs}^2 - F_{cal}^2\right)^2\right] / \left(N_{obs} - N_{par}\right)\right\}^{1/2}$

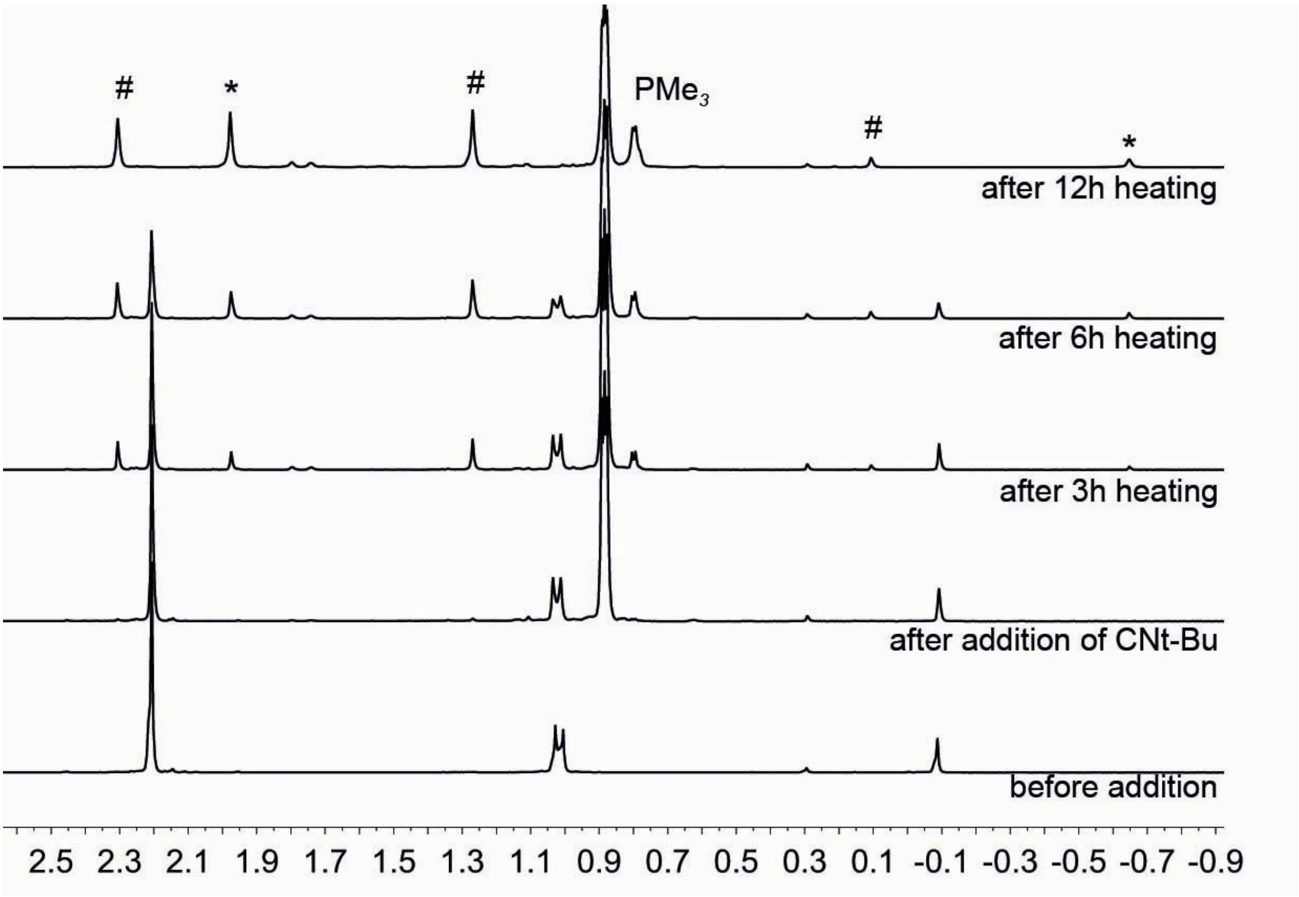

Figure S1: ^{1}H NMR reaction of **2** with an excess of NC^{t}Bu: resonances marked with an # represent signals from **3**, whereas resonances marked with an * represent signals from the side product Cp*ZnMe.

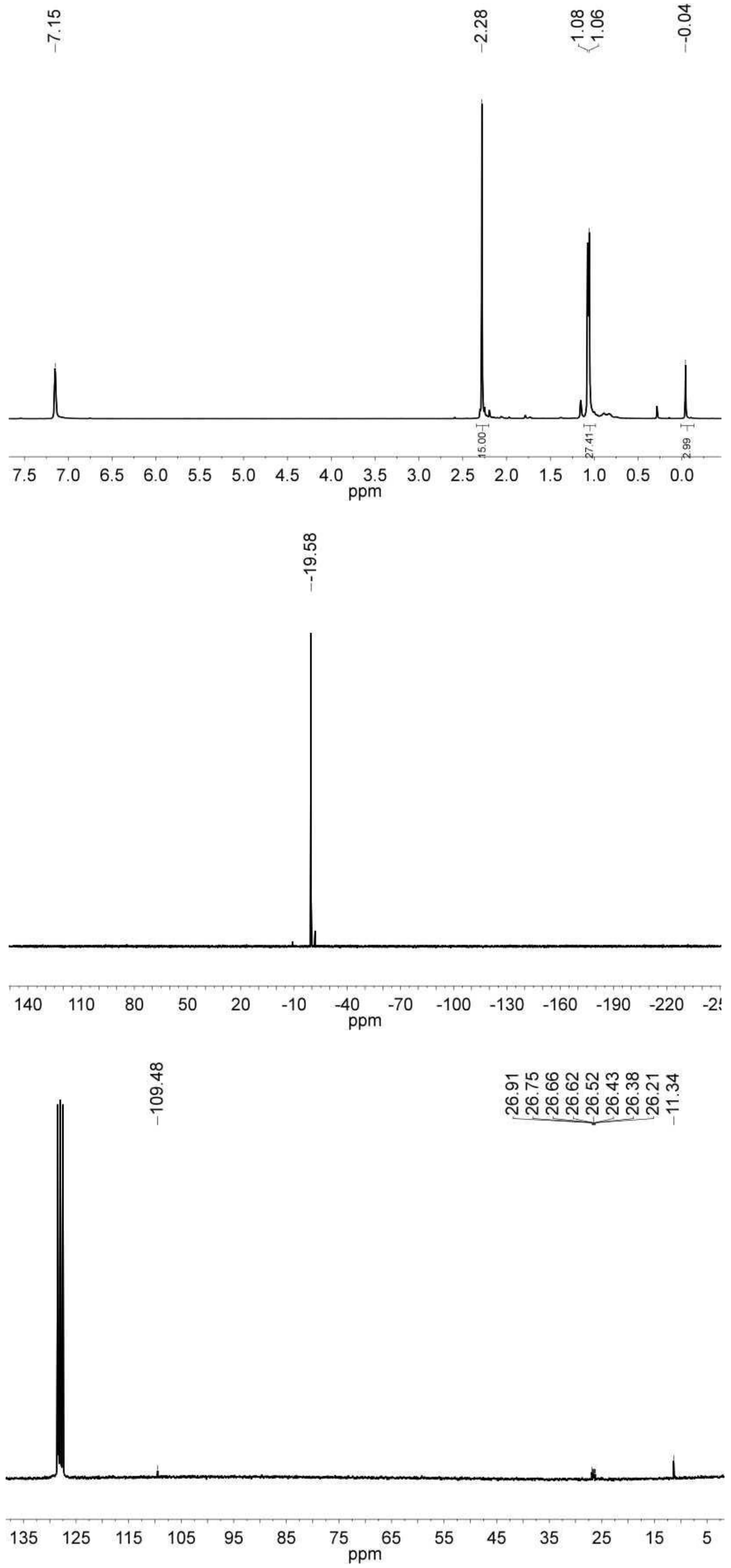

Figure S2. ^{1}H, ^{31}P and ^{13}C NMR spectra of **1**

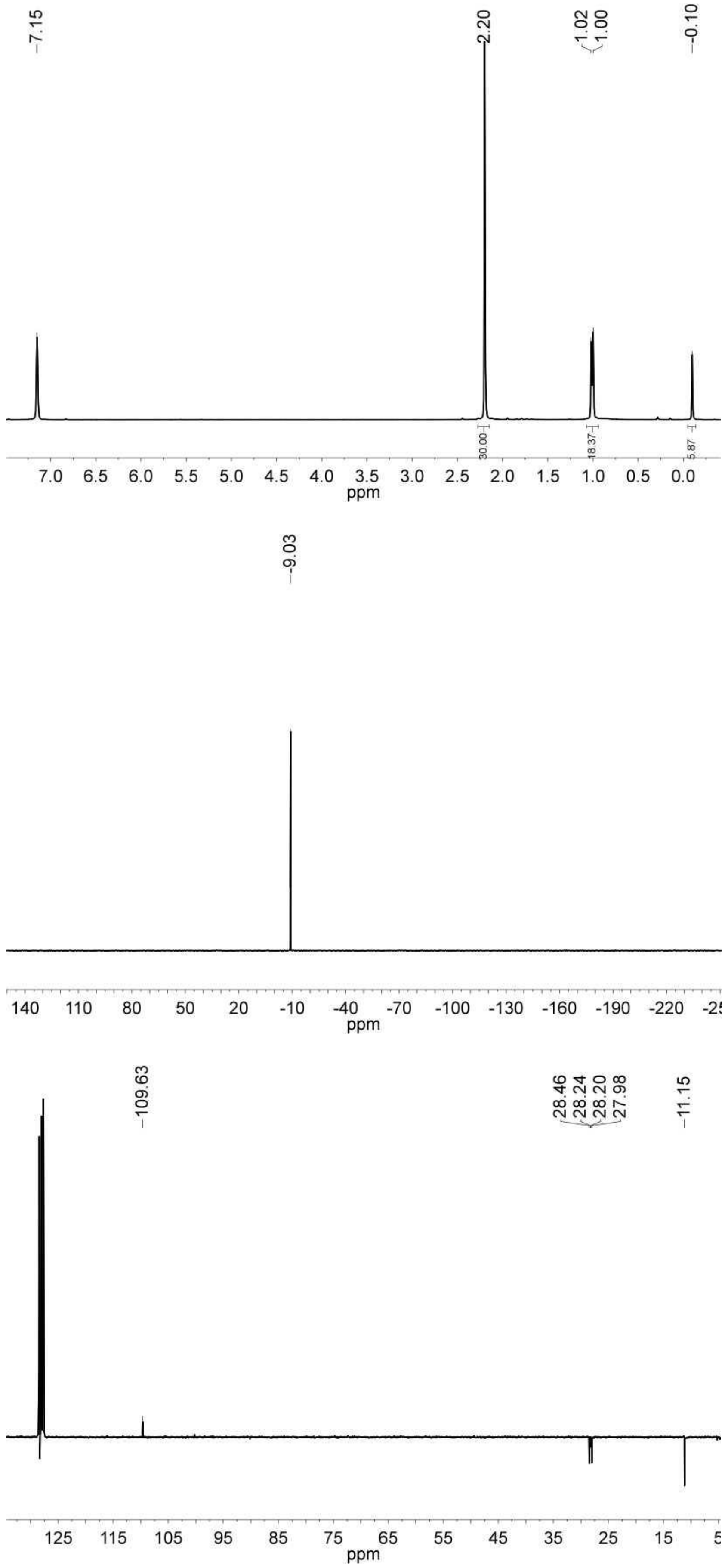

Figure S3. ^{1}H, ^{31}P and ^{13}C NMR spectra of **2**

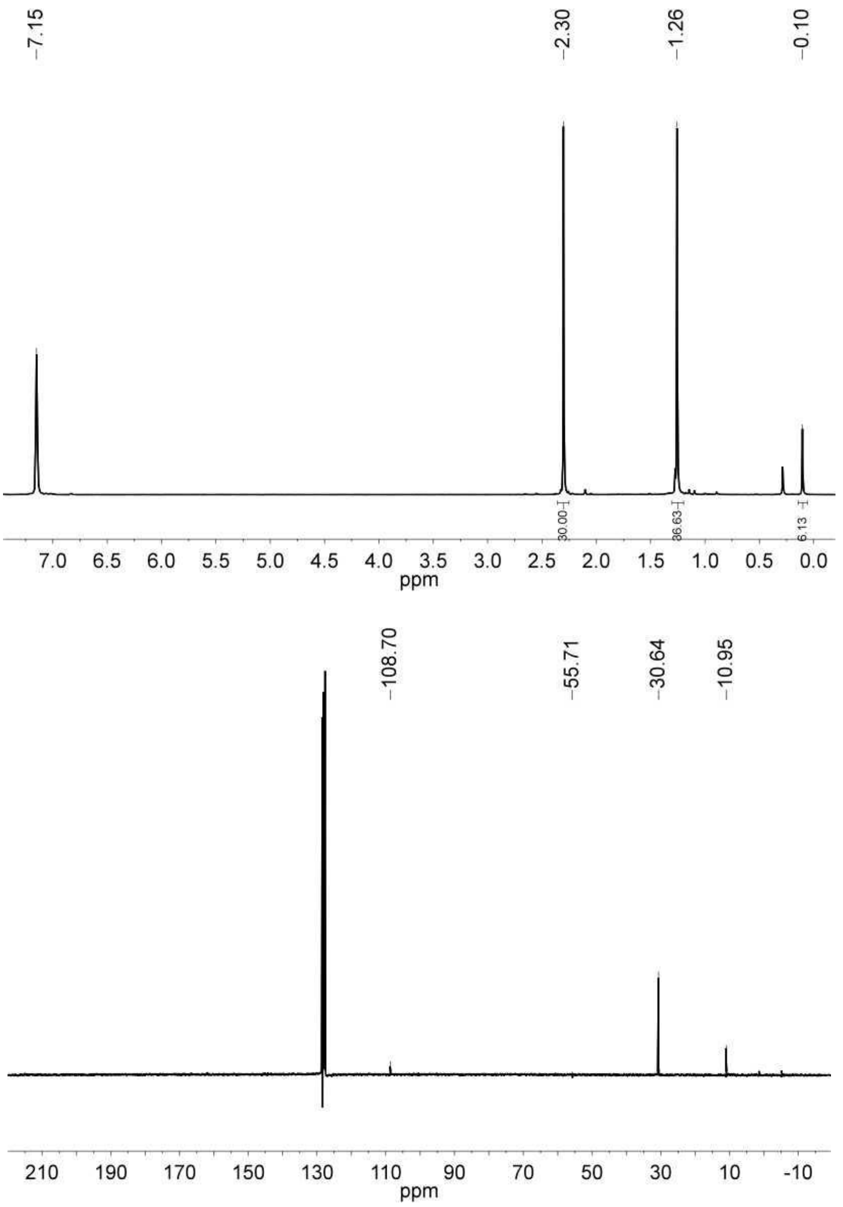

Figure S4. ^{1}H and ^{13}C NMR spectra of **3**

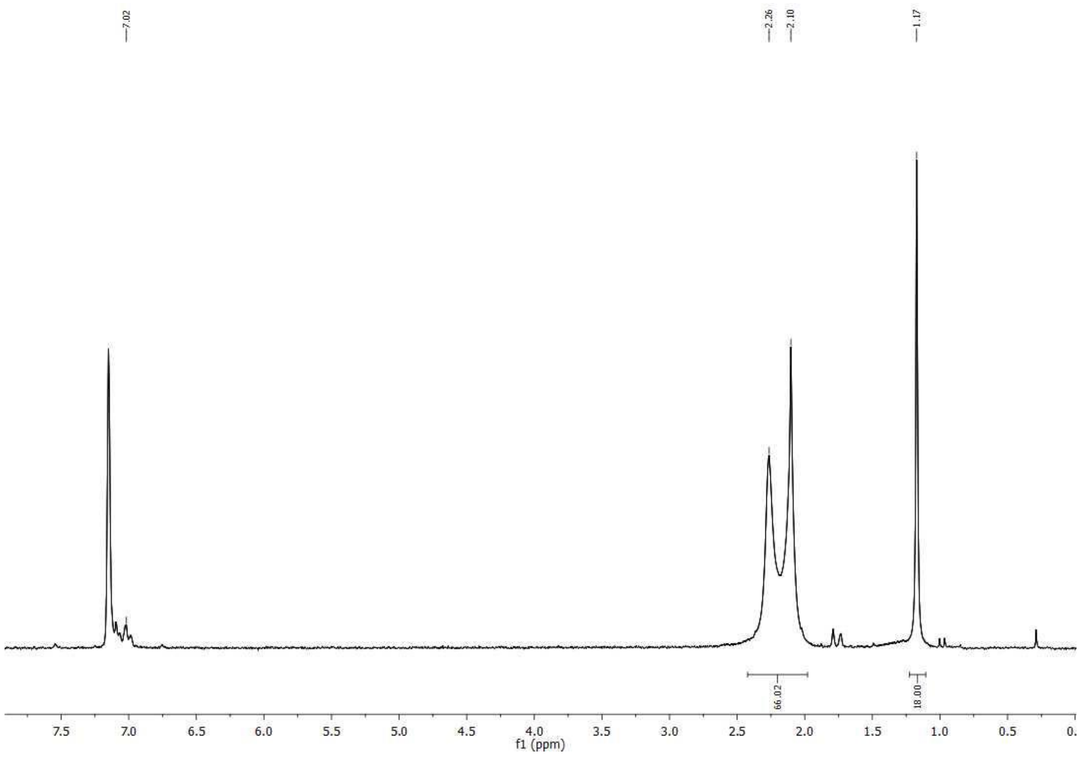

Figure S5. ^{1}H NMR spectra of **4**. Besides the product signals, signals for two toluene molecules, which co-crystallize, can be observed

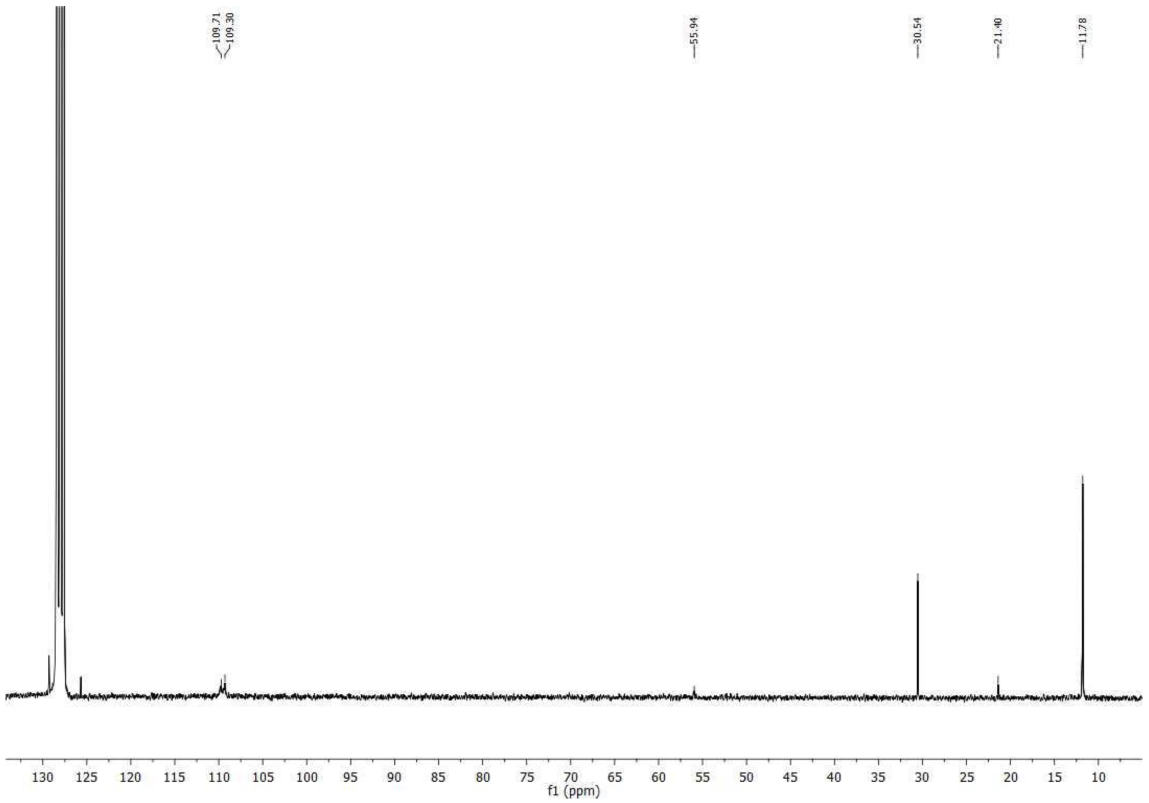

Figure S6. ^{13}C NMR spectra of **4**

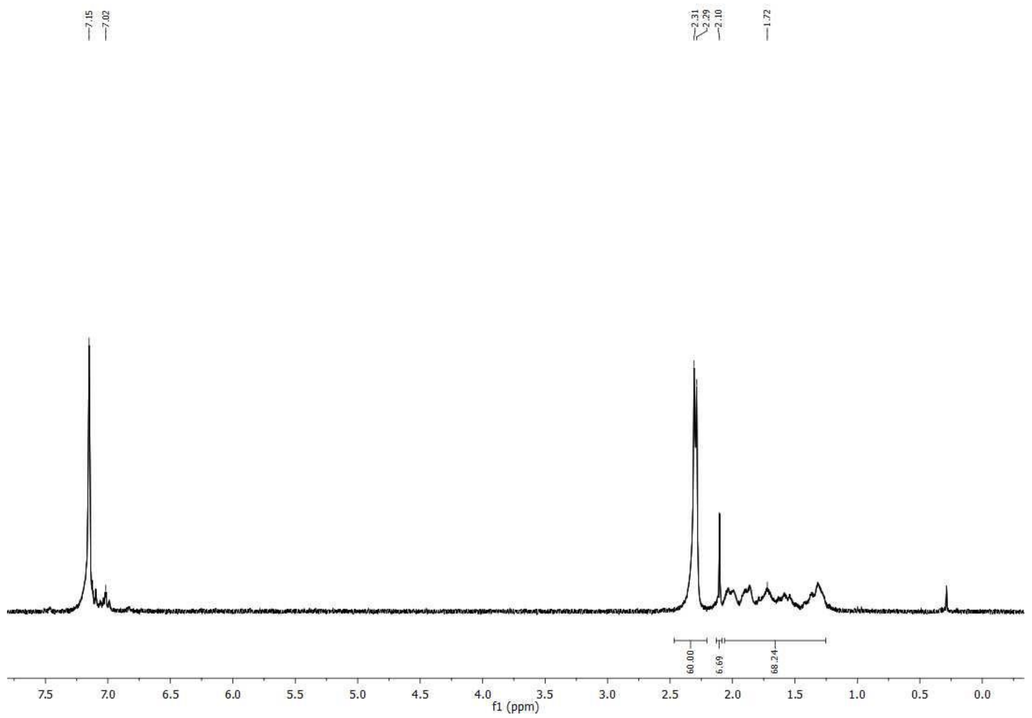

Figure S6. ^{1}H NMR spectra of **5**. Besides the product signals, signals for toluene which co-crystallize can be observed

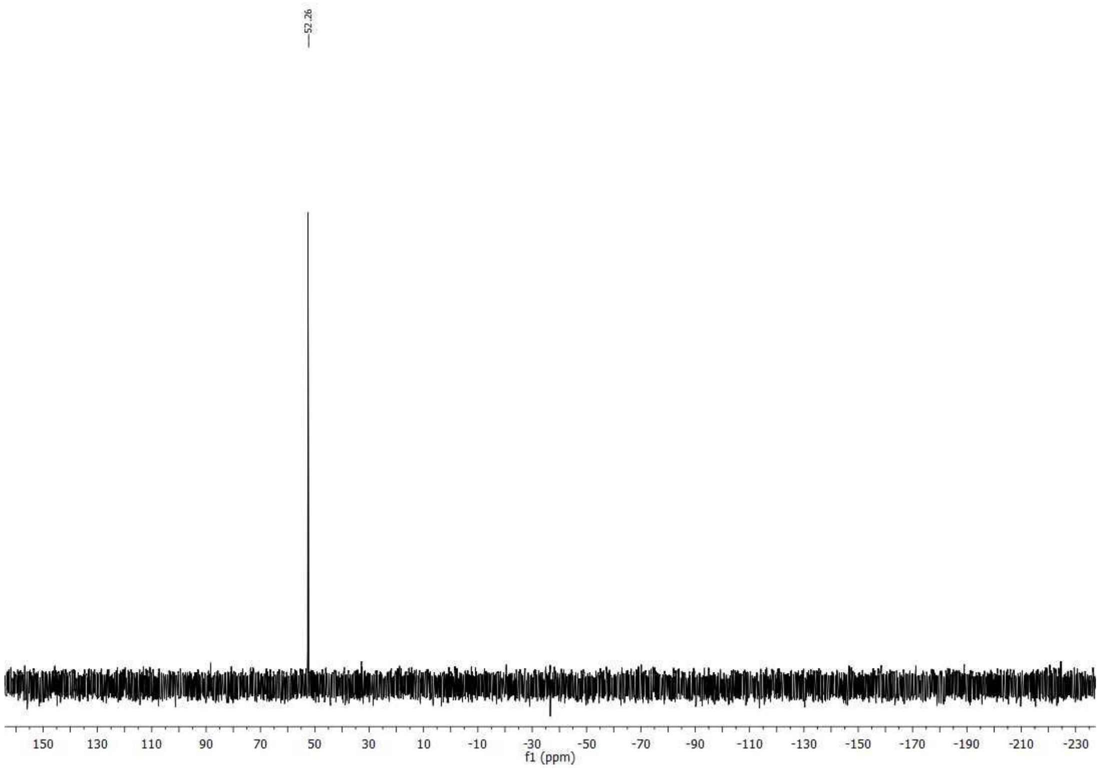

Figure S7. ^{31}P NMR of **5**

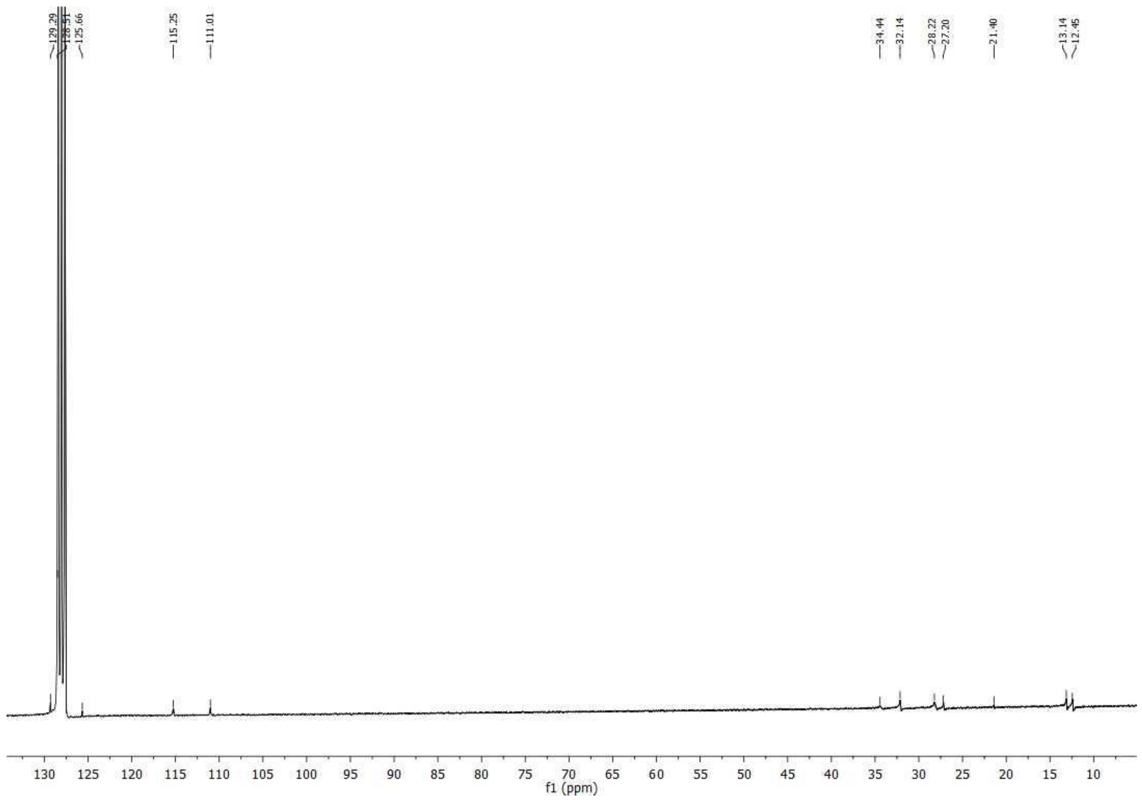

Figure S8. ^{13}C NMR spectra of **5**. Besides the product signals, signals for toluene which co-crystallize can be observed

Further Information on Continuous Shape Measure (CShM)

Continuous shape measure (CShM) is a mathematical method for the comparison of two different polyhedra. In this method, N vertices of a polyhedron are given by their position vectors Q_i (i = 1, 2, 3 ..., N), as well as N vertices of an second polyhedron with the position vectors P_i (i = 1, 2, 3 ..., N). The smallest distance $S_Q(P)$ of the position vectors between both polyhedrons is expressed with the following equation

$$S_Q(P) = \frac{1}{N} min \sum_{i=1}^{N} \left|\overline{Q_i} - P_i\right|^2 \cdot 100$$

The polyhedrons tested were centered in the origin and standardized ($|\vec{V}_i| = |\vec{V}_j| / \overline{|\vec{V}_j|}$) first. Final values of $0 \leq S_Q(P) \leq 100$ can be obtained, which serve as a quantitative measure for the analogousness of both polyhedra. With $S_Q(P) = 0$ the polyhedron represents exact overlap of both polyhedrons, while increasing values denote increasing distortions. In our approach, we have chosen a computer-aided method for finding the minimum distance, i.e. for identifying the best superimposition of both polyhedra: The two polyhedra with the transition metal centre in the origin were superimposed. One of the two polyhedra is then rotated around three independent axes by 360° in steps of 360/n degrees resulting in n^3 different superimpositions. For each step a “minimum distance” of the polyhedral vertices is calculated by permutation of all plausible vertex combinations. This procedure results in n^3 distance values, the smallest one representing the most ideal superimposition of the two polyhedra. For this superimposition, the shape measure $S_Q(P)$ is calculated as described above. Figure S9a to S9c show the most ideal superposition for the corresponding ideal polyhedrons and those extracted from molecular structures of **1** and **2**.

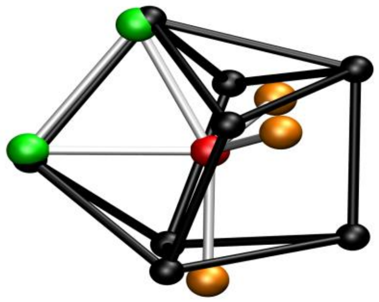

Figure S9a: Superimposition of a dodecahedron (black) and the fragment [$NiZn_2P_3$] extracted from the molecular structure of **1** (coloured), concerning Zn atoms

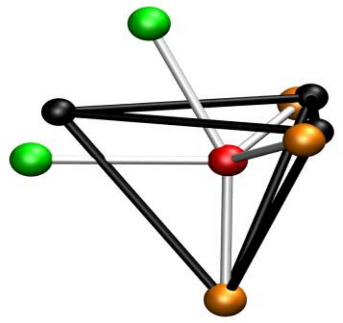

Figure S9b: Superimposition of a tetrahedron (black) and the fragment [$NiZn_2P_3$] extracted from the molecular structure of **1** (coloured), concerning P atoms

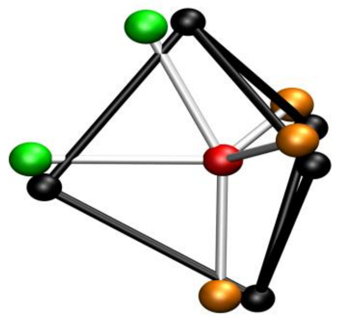

Figure S9c: Superimposition of a trigonalbipyramid (black) and the fragment [$NiZn_2P_3$] extracted from the molecular structure of **1** (coloured), concerning Zn and P atoms

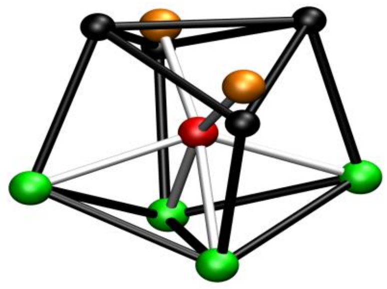

Figure S10a: Superimposition of a dodecahedron (black) and the fragment [$NiZn_4P_2$] extracted from the molecular structure of **2** (coloured), concerning Zn atoms

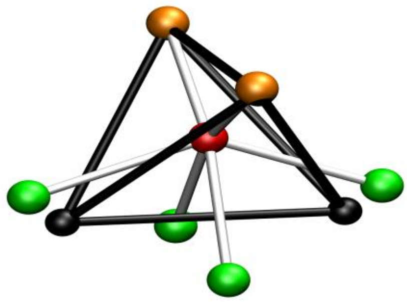

Figure S10b: Superimposition of a tetrahedron (black) and the fragment [$NiZn_4P_2$] extracted from the molecular structure of **2** (coloured), concerning P atoms

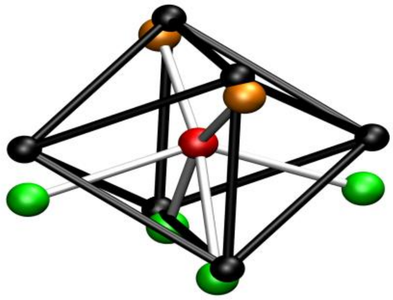

Figure S10c: Superimposition of a octahedron (black) and the fragment [$NiZn_4P_2$] extracted from the molecular structure of **2** (coloured), concerning Zn and P atoms

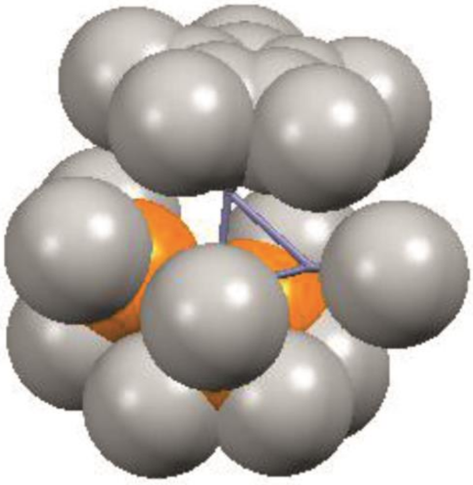

Figure S11: Space filling model for **1**.

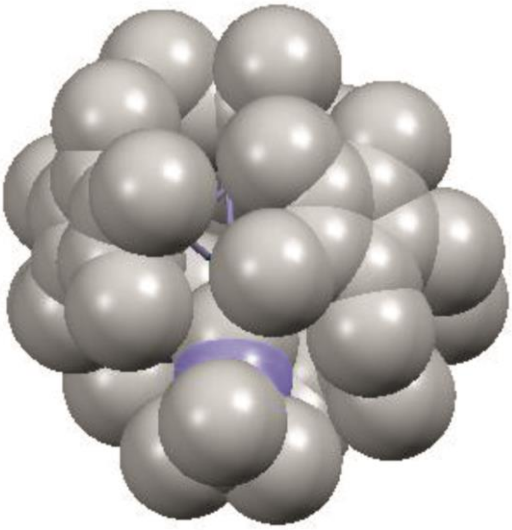

Figure S12: Space filling model for **4**.

Quantumchemical Calculations

Theoretical Methods:

The geometries of the molecules were optimized at the gradient corrected DFT level of theory using Becke's exchange functional[1] in conjunction with Perdew's correlation functional[2] (BP86) with the TURBOMOLE V6.3 program package.[3] Ahlrich's def2-TZVPP basis set [4] was used. The RI approximation[5] was applied using auxiliary basis functions.[6] Stationary points were characterized by the analytical calculation of the Hessian using TURBOMOLE's AOFORCE module.[7] The level of theory is then denoted as BP86/TZVPP.

The Atom-in-Molecules (QTAIM)[8] analyses were carried out with the program AIMAll[9] using the BP86/TZVPP wavefunction files.

The NBO charges were obtained using the NBO 3.1 program implemented in Gaussian09.[10],[11]

The EDA-NOCV calculations[16] were carried out using the ADF(2013.01)[12] program package at the BP86/TZ2P+ level of theory. Uncontracted Slater-type orbitals (STOs) were employed as basis functions in self-consistent field (SCF) calculations.[13] Triple-zeta-quality basis sets were used which were augmented by two sets of polarization functions, that is, p and d functions for the hydrogen atom and d and f functions for the other atoms. An auxiliary set of s, p, d, f and g STOs was used to fit the molecular densities and to represent the Coulomb and exchange potentials accurately in each SCF cycle.[14] Scalar relativistic effects were considered using the zero-order regular approximation (ZORA).[15]

Within the EDA, bond formation between the interacting fragments is divided into three steps: In the first step, the fragments which are calculated with the frozen geometry of the entire molecule, are superimposed without electronic relaxation to yield the quasiclassical electrostatic attraction $\Delta E_{\mathrm{elstat}}$. In the second step, the product wave function becomes antisymmetrized and renormalized, which gives the repulsive term $\Delta E_{\mathrm{Pauli}}$, named the Pauli repulsion. The third step consists of the relaxation of the molecular orbitals to their final form to yield to stabilizing orbital interaction ΔE_{orb}. The sum of the three terms $\Delta E_{\mathrm{elstat}}+ \Delta E_{\mathrm{Pauli}}+ \Delta E_{\mathrm{orb}}$ gives the total interaction energy ΔE_{int}. For further information we refer to the literature.[17]

References:

[1] A. D. Becke, *Phys. Rev. A* **1988**, *38*, 3098.

[2] J. P. Perdew, *Phys. Rev. B* **1986**, *33*, 8822.

[3] R. Ahlrichs, M. Bär, M. Häser, H. Horn, C. Kölmel, *Chem. Phys. Lett.* **1989**, *162*, 165.

[4] F. Weigend, R. Ahlrichs, *Phys. Chem. Chem. Phys.* **2005**, *7*, 3297.

[5] R. Ahlrichs, *Phys. Chem. Chem. Phys.* **2004,** *6*, 5119.

[6] a) K. Eichkorn, O. Treutler, M. Häser, R. Ahlrichs, *Chem. Phys. Lett.* **1995**, *242*, 652; b) K. Eichkorn, F. Weigend, O. Treutler, R. Ahlrichs, *Theor. Chem. Acc.* **1997**, *97*, 119; c) F. Weigend, *Phys. Chem. Chem. Phys.* **2006**, *8*, 1057.

[7] P. Deglmann, K. May, F. Furche, R. Ahlrichs, *Chem. Phys. Lett.* **2004**, *384*, 103.

[8] R. F. W. Bader, *Atoms in Molecules. A Quantum Theory* **1990**, Oxford Univ. Press, Oxford.

[9] AIMAll (Version 13.10.19), Todd A. Keith, TK Gristmill Software, Overland Park KS, USA,

2013 (aim.tkgristmill.com).

[10] Gaussian 09, Revision A.02, M. J. Frisch, G. W. Trucks, H. B. Schlegel, G. E. Scuseria, M. A. Robb, J. R. Cheeseman, G. Scalmani, V. Barone, B. Mennucci, G. A. Petersson, H. Nakatsuji, M. Caricato, X. Li, H. P. Hratchian, A. F. Izmaylov, J. Bloino, G. Zheng, J. L. Sonnenberg, M. Hada, M. Ehara, K. Toyota, R. Fukuda, J. Hasegawa, M. Ishida, T. Nakajima,Y. Honda, O. Kitao, H. Nakai, T. Vreven, J. A. Montgomery, Jr., J. E. Peralta, F. Ogliaro, M. Bearpark, J. J. Heyd, E. Brothers, K. N. Kudin, V. N. Staroverov, R. Kobayashi, J. Normand, K. Raghavachari, A. Rendell, J. C. Burant, S. S. Iyengar, J. Tomasi, M. Cossi, N. Rega, J. M. Millam, M. Klene, J. E. Knox, J. B. Cross, V. Bakken, C. Adamo, J. Jaramillo, R. Gomperts, R. E. Stratmann, O. Yazyev, A. J. Austin, R. Cammi, C. Pomelli, J. W. Ochterski, R. L. Martin, K. Morokuma, V. G. Zakrzewski, G. A. Voth, P. Salvador, J. J. Dannenberg, S. Dapprich, A. D. Daniels,O. Farkas, J. B. Foresman, J. V. Ortiz, J. Cioslowski, and D. J. Fox, Gaussian, Inc., Wallingford CT, **2009**.

[11] a) A. E. Reed, R. B. Weinstock, F. Weinhold, *J. Chem. Phys.*, **1985**, *83*, 735; b) A. E. Reed, L. A. Curtiss, F. Weinhold, *Chem. Rev.*, **1988**, *88*, 899-926.

[12] ADF2012.01, SCM, Theoretical Chemistry, Vrije Universiteit, Amsterdam, http://www.scm.com.

[13] J. G. Snijders, P. Vernooijs, E. J. Baerends, *Atomic Data and Nuclear Data Tables,* **1981**, *26*, 483-509.

[14] J. Krijn, E. J. Baerends, *Internal Report: Fit Functions in the HFSMethod*, Vrije Universiteit Amsterdam, **1984**.

[15] a) C. Chang, M. Pelissier, P. Durand, *Phys. Scr.*, **1986**, *34*, 394-404; b) J.-L. Heully, I. Lindgren, E. Lindroth, S. Lundqvist, A.-M. Martensson-Pendrill, *J. Phys. B*, **1986**, *19*, 2799; c) J. Snijders, *Chem. Phys. Lett.*, **1996**, *252*, 51-61; d) E. V. Lenthe, E. J. Baerends, J. G. Snijders, *J. Chem. Phys.*, **1993**, *99*, 4597; e) E. van Lenthe, R. van Leeuwen, E. J. Baerends, J. G. Snijders, *Int. J. Quantum Chem.*, **1996**, *57*, 281-293.

[16] a) T. Ziegler, A. Rauk, *Theor. Chem. Acc*. **1977**, *46*, 333. b) M. Mitoraj, A. Michalak, *J. Mol. Model*. **2007**, 13, 347. c) A. Michalak, M. Mitoraj, T. J. Ziegler, *Phys. Chem. A*. **2008**, 112 , 1933. d) M. P. Mitoraj, A. Michalak, T. Ziegler, *J. Chem. Theory Comput*. **2009**, *5*, 962.

[17] a) M. von Hopffgarten, G. Frenking, *WIREs Comput. Mol. Sci.* **2012**, *2*, 43. b) M. Lein, G. Frenking, in Theory and Applications of Computational Chemistry (Eds.: E. D. Clifford, G. Frenking, S. K. Kwang, E. S. Gustavo), Elsevier, Amsterdam, 2005, pp. 291; b) G. Frenking, K. Wichmann, N. Fröhlich, C. Loschen, M. Lein, J. Frunzke, V. M. Rayon, *Coord. Chem. Rev*. **2003**, *238*, 55. d) G. Frenking, F.M. Bickelhaupt, *The Chemical Bond. 1. Fundamental Aspects of Chemical Bonding*, p. 121-158, G. Frenking and S. Shaik (Eds), Wiley-VCH, Weinheim, 2014. F. M. Bickelhaupt and E. J. Baerends, *Rev. Comput. Chem*, **2000**, *15*, 1.

Table S2: NBO results for **1**, [(ZnCp*)(ZnMe)] and **2** (BP86/def2-TZVPP).

System	Partial Charge [*e*]	Wiberg Bond Index
**1**		Zn-Zn: 0.26
ZnMe	+0.31	Zn_{Me}-Ni: 0.20
ZnCp*	-0.06	Zn_{Cp*}-Ni: 0.28
Ni	-0.23	
**[(ZnCp*)(ZnMe)]**		Zn-Zn: 0.65
ZnMe	+0.17	-
ZnCp*	-0.17	-
**2**		Zn-Zn: 0.07-0.15
ZnMe	+0.32	Zn_{Me}-Ni: 0.51
ZnCp*	+0.31	Zn_{Cp*}-Ni: 0.58
Ni	-2.37	

Table S3: EDA-NOCV results in kcal/mol for **1** (BP86/TZ2P+). Occupation of the fragments: [(ZnCp*)(ZnMe)]: singlet ; [Ni(PMe_3)$_3$] : singlet.

ΔE_{int}	-49.8	
ΔE_{Pauli}	165.6	
ΔE_{elstat}	-155.3 (72.1%)	
ΔE_{orb}	-60.1 (27.9%)	
$\Delta E_{orb,(1)}$	-27.7 (46.1%)	Ni→Zn_2
$\Delta E_{orb,(2)}$	-17.0 (28.3%)	Zn_2→Ni
$\Delta E_{orb, rest}$	-15.4 (25.6%)	Polarisation

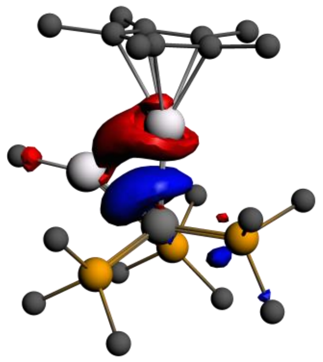	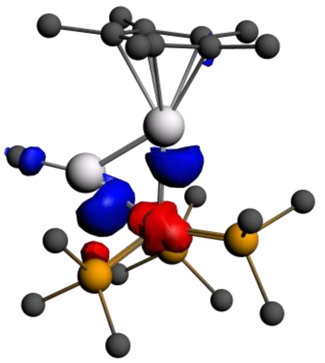
v= 0.448, ΔE_{orb} = -17.0 kcal/mol	v = 0.568, ΔE_{orb} = -27.7 kcal/mol

Table S4: EDA-NOCV results in kcal/mol for **2** (BP86/TZ2P+). Occupation of the fragments: ZnMe: doublet; $[Ni(ZnCp^*)_2(ZnMe)(PMe_3)_2]$: doublet.

ΔE_{int}	-69.4	
ΔE_{Pauli}	209.5	
ΔE_{elstat}	-171.8 (61.6%)	
ΔE_{orb}	-107.1 (38.4%)	
$\Delta E_{orb,(1)}$	-79.2 (73.9%)	Zn→Ni
$\Delta E_{orb,(2)}$	-10.6 (9.9%)	Ni→Zn
$\Delta E_{orb, rest}$	-17.3 (16.2%)	Polarisation

Table S5: EDA-NOCV results in kcal/mol for **2** (BP86/TZ2P+). Occupation of the fragments: ZnCp: doublet; $[Ni(ZnCp^*)(ZnMe)_2(PMe_3)_2]$: doublet.

ΔE_{int}	-67.0	
ΔE_{Pauli}	139.5	
ΔE_{elstat}	-137.4 (66.5%)	
ΔE_{orb}	-69.1 (33.5%)	
$\Delta E_{orb,(1)}$	-53.9 (78.0%)	Zn→Ni
$\Delta E_{orb,(2)}$	-3.8 (5.5%)	Ni→Zn
$\Delta E_{orb, rest}$	-11.4 (16.5%)	Polarisation

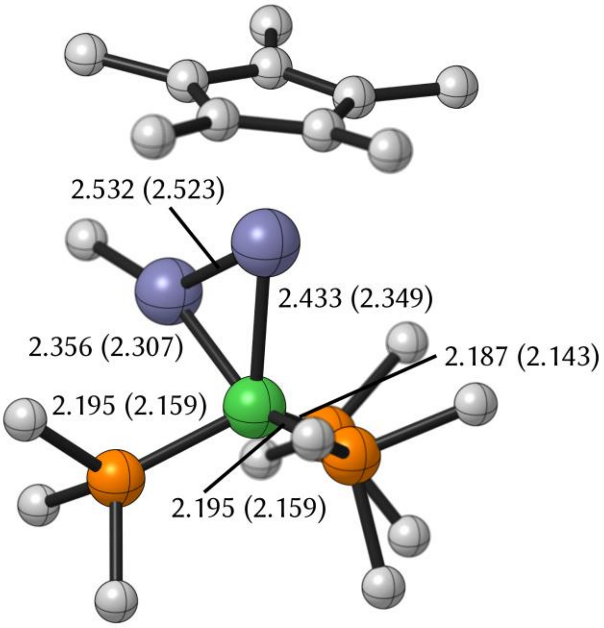

Figure S13: Calculated bond lengths [in Å] and angles [in °] of **1** (BP86/TZVPP). Experimental values are given in parentheses. Hydrogen atoms are omitted for a better view

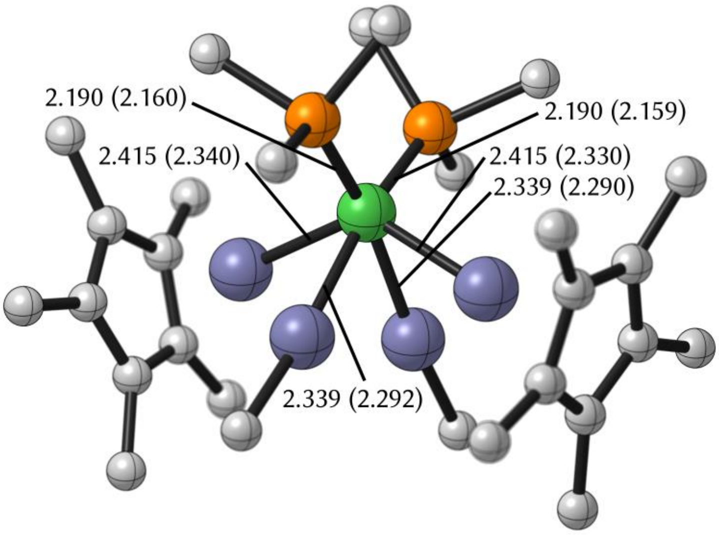

Figure S14: Calculated bond lengths [in Å] and angles [in °] of **2** (BP86/TZVPP). Experimental values are given in parentheses. Hydrogen atoms are omitted for a better view.
